# Effects of *Yersinia pseudotuberculosis* outer membrane vesicles on *Pseudomonas aeruginosa* antigens immune response

**DOI:** 10.1371/journal.pone.0310652

**Published:** 2024-12-20

**Authors:** Zhongxu Duan, Jingqi Song, Mingru Zhang, Zhe Zhang, Nan Li, Yuqin Fu, Zhe Sun, Tiancheng Lu, Siyuan Li, Mingyue Cao, Qingyu Wang, Chunhui Sun, Xiuran Wang

**Affiliations:** 1 Engineering Research Center of Bioreactor and Drug Development, Ministry of Education, College of Life Sciences, Jilin Agricultural University, Changchun, China; 2 State Key Laboratory of Black Soils Conservation and Utilization, Key Laboratory of Wetland Ecology and Environment Northeast Institute of Geography and Agroecology, Chinese Academy of Sciences, Changchun, China; 3 Changchun Veterinary Research Institute, Chinese Academy of Agricultural Sciences, Beijing, China; 4 Research Center, The Seventh Affiliated Hospital of Sun Yat-Sen University, Shenzhen, China; 5 School of Laboratory Animal & Shandong Laboratory Animal Center, Shandong First Medical University & Shandong Academy of Medical Sciences, Huaiyin District, Jinan, China; Universidad Nacional de la Plata, ARGENTINA

## Abstract

Outer membrane vesicles (OMVs) are immunogenic self-adjuvanting vesicles produced by Gram-negative bacteria such as *Pseudomonas aeruginosa* and *Yersinia pseudotuberculosis*. While the effects of OMVs on different antigens immune stimulation are not clear. In this study, we constructed recombinant *Yersinia pseudotuberculosis ΔlpxL* strain,with *pBlue-PcrV* and *pBlue-OprF/I*, and then purified *ΔlpxL* rOMV_PcrV_ (rOMVyp2P)and *ΔlpxL* rOMV_OprF/I_ (rOMVyp2F) and analyzed its effect on immune response and protection against *Pseudomonas aeruginosa* PAO1 infection. The results showed that OMV assists in eliciting similar humoral immune responses to PcrV and OprF/I antigens. *ΔlpxL* rOMV_PcrV_ and *ΔlpxL* rOMV_OprF/I_ elicited Th1/Th2 balanced immune response, and higher IgM and IgA antibodies.However, there are differences in immune protection for the pulmonary. The survival rate of mice in *ΔlpxL* rOMV_PcrV_ group was 20%, which was significantly better than that in *ΔlpxL* rOMV_OprF/I_ group. *ΔlpxL* OMV_PcrV_ is better cooperation for *Pseudomonas* immune protection in lung.

## 1. Introduction

Outer membrane vesicles (OMVs) are spherical buds produced by the outer membrane of Gram-negative bacteria with diameters ranging from 20 to 250 nm **[1]**. It has been found that OMVs are mainly composed of lipopolysaccharides, proteins (including various enzymes), peptidoglycan, DNA, RNA, and other small molecules **[2]**. OMVs have been demonstrated to be involved in numerous physiological processes, including protein transport, nutrient acquisition, intercellular communication, antimicrobial activity, toxin transport, and the modulation of host immune responses. **[3]**.

In recent years, the research of OMVs on vaccines is in full swing. The recombinant OMVs are the majority protocol for vaccine construction, while the selection of antigens and the effect of OMVs on different antigenic proteins should be spell out during the construction. OMVs can be derived from different bacteria, and different species of OMVs may have different biological activities **[4,5]**. The immune response of same antigens with different OMVs is similar, but the effect of OMVs on different antigens reported scarecely **[6]**. OMVs have functions such as signaling and genetic material transfer activity, which may influence the mode of response between OMVs and the target proteins. Additionally, characteristics of the target proteins, such as size, charge, and affinity, can impact their interaction with OMVs **[7,8]**. The biological environment also affects OMVs and target proteins, such as pH, ion concentration, temperature, and other factors. The concentration and size of OMVs also affect their interactions with target proteins. OMVs can also react with various types of cells, including immune and epithelial cells **[9–12]**. Besides, protein antigens in OMVs may be processed by proteases during fusion with phagocytic vesicles, which in turn release delivery to MHC II molecules to activate CD4+ T-cell immunity **[13,14]**. OMVs can be captured by Dendritic Cells (DCs) and transported through lymphatic vessels to lymph nodes. During this process, DCs process the recombinant OMVs, resulting in the production of effector cytokines, ultimately activating the immune response **[15,16]**. Differences in the composition of OMVs may be the main reason for the variations in immune protection **[17]**.

*Pseudomonas aeruginosa* is an important Gram-negative opportunistic pathogen that causes infections. Patients who can cause trauma, severe pneumonia may need to be intubated (ventilator-associated pneumonia), or urinary tract infections may be severe enough to require catheter insertion. It is widely distributed in the external environment (stagnant freshwater) and specific reservoirs in the hospital environment (e.g., sink drains). In recent years, the irrational use of antimicrobial drugs has led to P. aeruginosa developing multidrug resistance and other concerning traits, making its infections a significant challenge in current clinical anti-infective therapies in hospitals. Therefore, vaccine development is the best way to prevent infection **[18]**.

It has been found that *P*. *aeruginosa* vaccines are usually based on antigenic candidates, such as LPS O-antigen, alginate, flagellin, PcrV, PopB, OprF/I proteins. The PcrV protein, a prominent protective antigenic candidate, is a structural component of the type III secretion system (T3SS). It has also been identified as an ideal target for immunoprophylaxis and therapeutic studies **[19,20]**. It has been found that the use of PcrV as a protective protein antigen has been shown to provide a degree of protection in mice in the presence of other adjuvants (e.g., CpG, curdlan, and alum). However, subunit vaccines currently remain somewhat problematic in that, while they are able to produce specific antibodies and reduce bacterial loading of the liver in mice, they are not significantly efficacious in terms of bacterial clearance, and fail to improve patient morbidity significantly, as a result of which no mature *P*. *aeruginosa* vaccine has been introduced so far **[21–24]**. PcrV and OprF/I are immunoprotective proteins selected from *P*. *aeruginosa*. Studies have shown that both PcrV and OprF/I were used as ideal candidate antigens for *P*. *aeruginosa* vaccine in clinical trial **[23]**.

Extensive research has provided growing evidence that immunizing mice with recombinant OMVs against heterologous antigens induces a protective response against infections by pathogens with these antigens. One study found that recombinant *Y*. *pseudotuberculosis* OMVs could be used as a vaccine delivery system to deliver the *Y*. *pestis* LcrV antigen **[25,26]**. The results showed that the recombinant OMV vaccine provided excellent protection against *bubonic plague and pneumonic plague* and intramuscular immunization of mice with *P*. *aeruginosa* PcrV-HitA recombinant OMV vaccine provided significant protection against the PA103 strain and stimulated strong specific B and T-cell responses **[27]**. Therefore, this system is feasible for the delivery *of P*. *aeruginosa* PcrV antigens to prevent *P*. *aeruginosa* infections. Although OMVs can produce good immune protection and can be used as a novel vaccine delivery system, they still have some toxicity. *Y*. *pseudotuberculosis* lipid A is the main source of toxicity, and the genes code lipid A are almost clear. It was reported the clearance of lipopolysaccharides can reduce toxicity, but it may also lead to a decrease in the ability of OMV to activate the immune system. Acyltransferases *lpxL* mutant will cause lower OMVs toxicity. *lpxA* code UDP-N-acetylglucosamine acyltransferase, but its influence on OMV immune activation is still unclear. and it can also be used as a delivery system for heterologous antigens, which has gained wide attention in the research of OMV vaccine **[28–31].**

OMV as a self-adjuvant in vaccine development has attracted more and more attention. Are there some differences of OMVs combined different antigens? In this study, we prepared PcrV and OprF/I antigens to evaluate the effects of OMVs on these antigens’ immunity and protection. If it is possible, we can cooperation different sources of strong candidates together in future.

## 2. Materials and methods

### 2.1 Strains and plasmids

**Table 1** lists all the strains and plasmids used in this study. *Y*. *pseudotuberculosis* YP0 was cultured in LB liquid medium at 28°C.On the other hand *P*. *aeruginosa* PAO1 and *E*. *coli BL21(DE3)* were cultured in LB liquid medium at 37°C.

**Table 1:** Strains and plasmids used in this study.

Strain or plasmid	Genotype or relevant characteristics	Source or reference
*YP0*	*Yersinia pseudotuberculosis GDMCC1*.*267*	*Received from Institute of Microbiology*, *Guangdong Academy of Sciences*
*Yp0P*	*Yersinia pseudotuberculosis GDMCC1*.*267*pNP214*-PcrV*	*Lab constructed*
*Yp1P*	*Yersinia pseudotuberculosis GDMCC1*.*267*:: *ΔLpxA*::pNP214*-PcrV*	*Lab constructed*
*Yp2P*	*Yersinia pseudotuberculosis GDMCC1*.*267*:: *ΔLpxL*::pNP214*-PcrV*	*Lab constructed*
PAO1	*Pseudomonas aeruginosa* SHBCC D24625	*Received from Shanghai Bioresource*
*E*.*coliBL21(DE3)*	pNP214*-PcrV*	*Lab constructed*
Plasmids		*Purchased from the Miao Ling plasmid platform*
Pre112-*Δ*lpxL	SM10λPir (+)	*Lab constructed*
Pre112-*Δ*lpxA	SM10λPir (+)	*Lab constructed*
pNP214*-PcrV*	pBuescriptSKII (+)	*Lab constructed*
pNP214*-OprF/I*	pBuescriptSKII (+)	*Lab constructed*

### 2.2 Yp1 and Yp2 strain construction

The specific steps of strain construction are as follows: Based on pRE112 plasmid, we designed and synthesized homologous arm primers at both ends of two genes segments (*LpxA*,*LpxL*). Gene fragments were cloned by PCR, and the suicide plasmid was constructed by homologous recombination principle., and then transferred the recombinant plasmid into *Y*. *pseudotuberculosis* YP0 by electrical conversion method. The positive strains were confirmed by three repeated antibiotic screening (LB agar contained 50μg/mL Cm) and PCR identification. The mutant strains were finally identified by sucrose screening (LB agar with 10% sucrose).

### 2.3 Preparation of recombinant OMVs from *Y*. *pseudotuberculosis* by extraction

rOMVyp0P (OMV_PcrV_) originating from the Yp0P strain, rOMVyp1P (*ΔlpxA* OMV_PcrV_) from the Yp1P strain, rOMVyp2P (*ΔlpxL* OMV_PcrV_) Yp2P strain and rOMVyp2F/I (*ΔlpxL* OMV_OprF/I_) from the Yp2F/I strain were extracted using ultrafiltration centrifugation. The specific operation steps are as follows: Yp0P, Yp1P, Yp2P and Yp2F/I strains were inoculated on LB agar plates and cultured overnight at 28°C. The single colony cultured for 16 h was transferred to a 5-mL LB liquid medium and incubated at 28°C at 200 rpm until the logarithmic growth period, then transferred to a 1-L LB liquid medium. When the OD600 value reached 0.4–0.6, 0.5M EDTA was added, and the solution was incubated on ice for 1 hour to prepare for the extraction of recombinant OMVs. The procedure was as follows: the bacterial suspension was centrifuged at 10,000 × g for 15 minutes at 4°C, and the supernatant was collected. The supernatant was then filtered through a 0.22-μm filter to remove bacteria. The filtered solution was concentrated to 40 mL using a cyclical process. This was followed by ultracentrifugation at 120,000 × g, 4°C for 2 hours. The supernatant was discarded, and the pellet was resuspended in a small volume of sterile 1/10× PBS. The sample was then centrifuged at 4,000 × g for 1 minute, filtered through a 0.22-μm aqueous phase filter membrane, collected in a sterile 1.5-mL centrifuge tube, and stored at 4°C.The recombinant OMV was subjected to SDS-PAGE electrophoresis, and the strips were cut and stored respectively. After digestion by enzyme solution and ammonium bicarbonate, mass spectrometry was used to detect the OMV. The false positive proteins were screened with pvalue and then verified on NCBI and the subcellular localization of the proteins was predicted by Gneg-mPLoc software.

### 2.4 Recombinant OMV particle size determination

First, the samples were treated with glutaraldehyde and dropped on copper mesh, negatively stained with 1–2% phosphotungstic acid (PTA, pH6.5–7.0), and dried for 5-10s. The samples were then observed by JEM-1200EXII transmission electron microscopy (80kv, 25000 X) **[26]**. The particle size and concentration of the sample were determined using a nanoparticle tracking analyzer. The sample was diluted 100-fold before being added to the analyzer, and the scanning computer control system was utilized for detection.

### 2.5 Feeding and management of mice

8-week-old male and female Balb/c mice with SPF grade were selected and divided into groups and each group 10 mice (n = 10, 5 male and 5 female). The mice were raised in the animal facility of Jilin GENET-MED Biotechnology Co., LTD. The feeding conditions of all mice were ensured to be consistent.

Ethical Statement: In this study, we strictly followed the Experimental Animal Center, Jilin Agricultural University Experimental Animal License: SYXK(Ji)2023-0021)and GB/T 35892–2018 Experimental Animal Welfare Ethical Review Guidelines. On day 28 after immunization, the mice were infected with *Pseudomonas aeruginosa* PAO1. There are two groups: subcutaneous and intranasal infections. Subcutaneous infection: 2x10^8^ CFU PAO1 in 100 μL PBS was injected directly under subcutaneous. Intranasal infection: Isoflurane with Fresh gas flow at 4L/min was used for general anesthesia, followed by 2x10^7^ CFU in 40μL PBS nasal drops to infect mice. On day 44, the surviving mice after two kinds of infection was euthanized with carbon dioxide to minimize the animals’ suffering.

### 2.6 Immunoprotection evaluation

The mice were immunized by intramuscular injection two times, on day 0 and day 21 respectively. The mice were immunized with 40μg/100μl rOMV and 5μg/100μl protein, respectively, and blood samples were collected by submaxillary venipentesis during immunization. Each group of 10 mice was divided into the following groups: 5 μg PcrV protein with aluminum hydroxide adjuvant, 40 μg rOMVyp0P, 40 μg rOMVyp1P, 40 μg rOMVyp2P, 40 μg OMVyp0, 5 μg OprF/I protein with aluminum hydroxide adjuvant, 40 μg rOMVyp2F/I, 40 μg rOMVyp2 and PBS blank control. After 42d immunization, 10LD50 PAO1 strain was used to evaluate the immune protection through subcutaneous (2×10^8^ CFU) and nasal infection (2×10^7^ CFU).

### 2.7 Determination of antibody levels

Mice were subjected to enhanced immunization 21 days after the initial immunization, blood samples were collected by submaxillary venipentesis 14 and 28 days after immunization, and serum was separated, and serum was used for ELISA to detect IgG, IgG1, IgG2a, IgM, and IgA antibody levels. The ELISA method was based on reference **[25]** for the experiment.

### 2.8 Cytokine determination

Changes in IFN-γ, IL-1β, IL-17A, IL-6, and TNF-α levels in mouse serum at 14 d were measured by ELISA. Test according to ELISA KIT instructions (purchased from Shanghai Jining Industrial Co., Ltd.).

### 2.9 Data analysis

All data are described as mean ± standard deviation of the three samples. Data was analyzed and summarized by Microsoft Excel 2019. Draw graphics with Graph Pad Prism. Survival analysis was performed using the log-rank (Mantel-Cox) test. Data are expressed as mean ± standard deviation (SD). ns, not significant; *, P<0.05; **, P<0.01; ***, P<0.001; ****, P<0.0001.

## 3. Results

### 3.1 Successful expression of *PcrV* and *OprF/I* in recombinant OMVs

**Fig 1:**
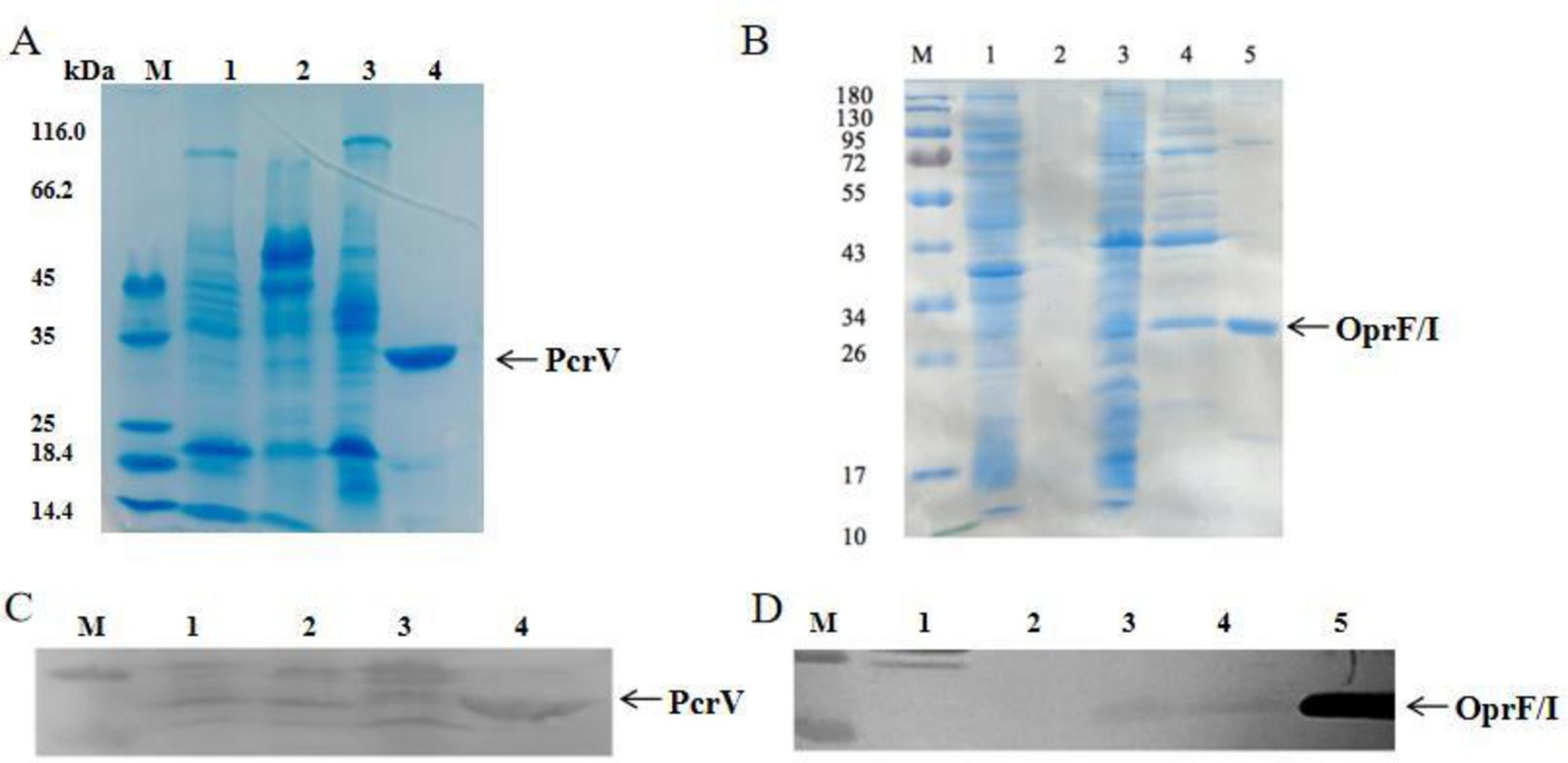
SDS-PAGE and Western blot were used to detect the expression of target protein in rOMV.

**Fig 2:**
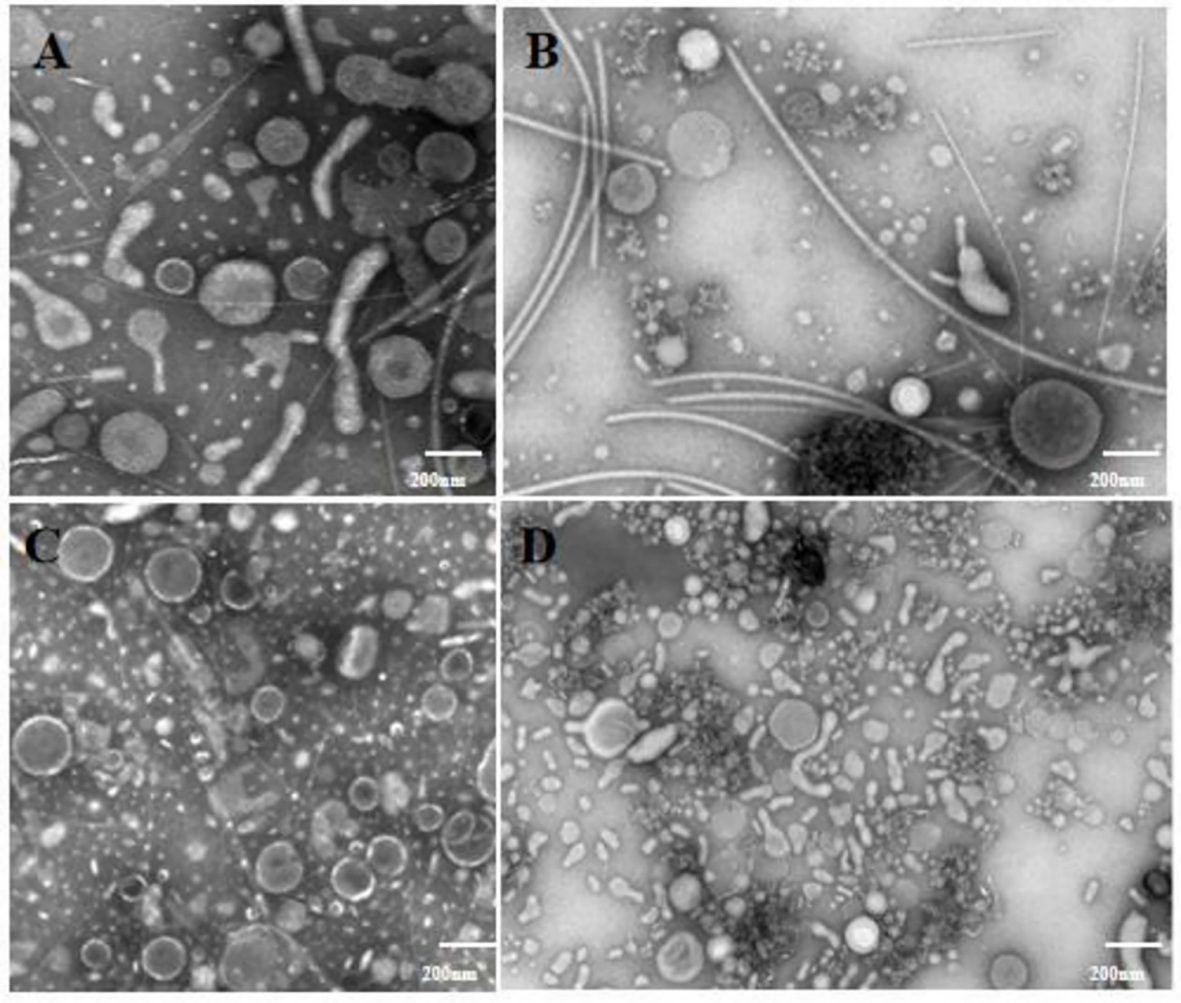
The diameter of the recombinant OMV was about 100-200nm observed by transmission electron microscopy.

**Fig 3:**
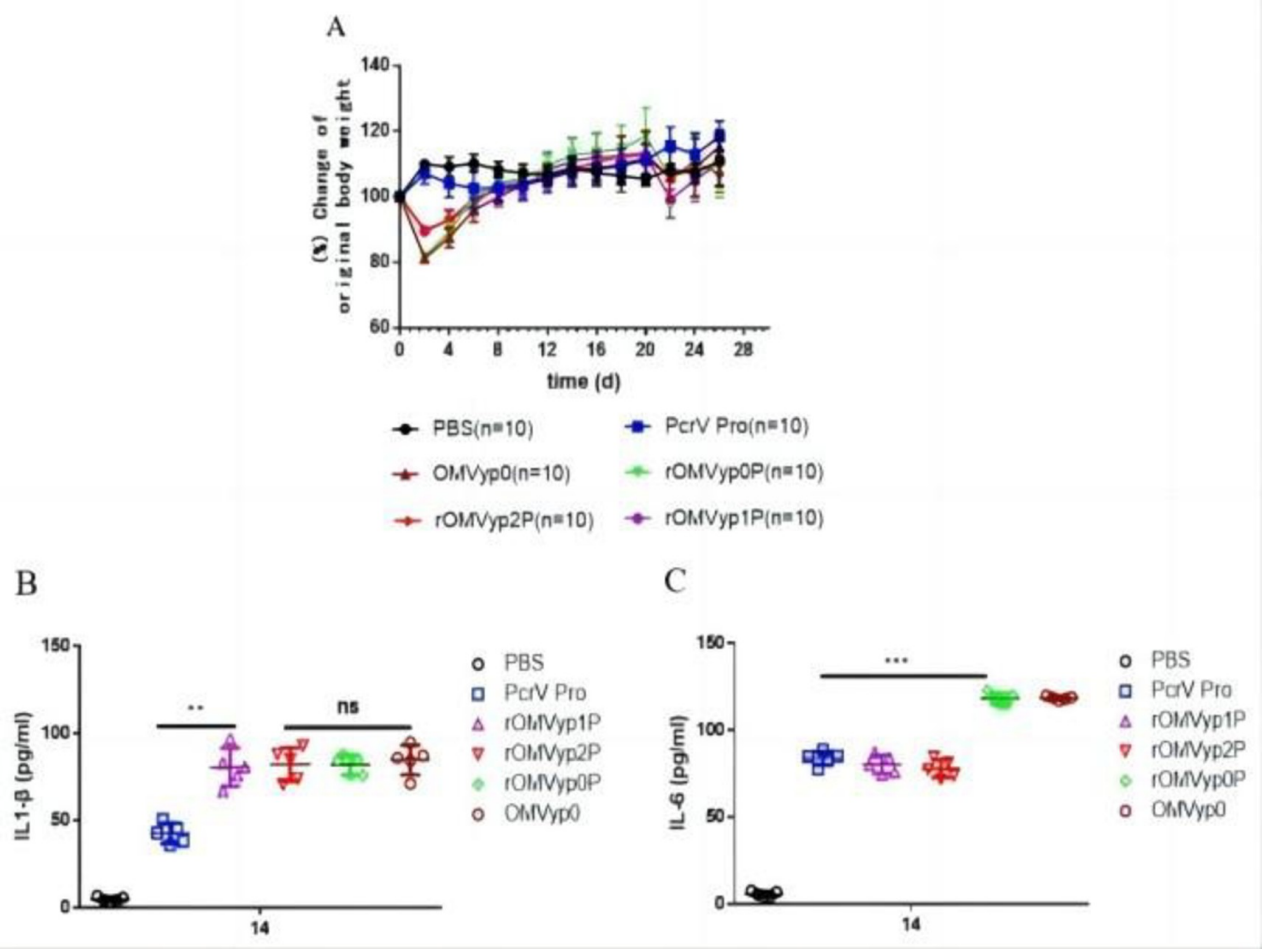
Changes of body weight and cytokines in rOMV_PcrV_ group.

**Fig 4:**
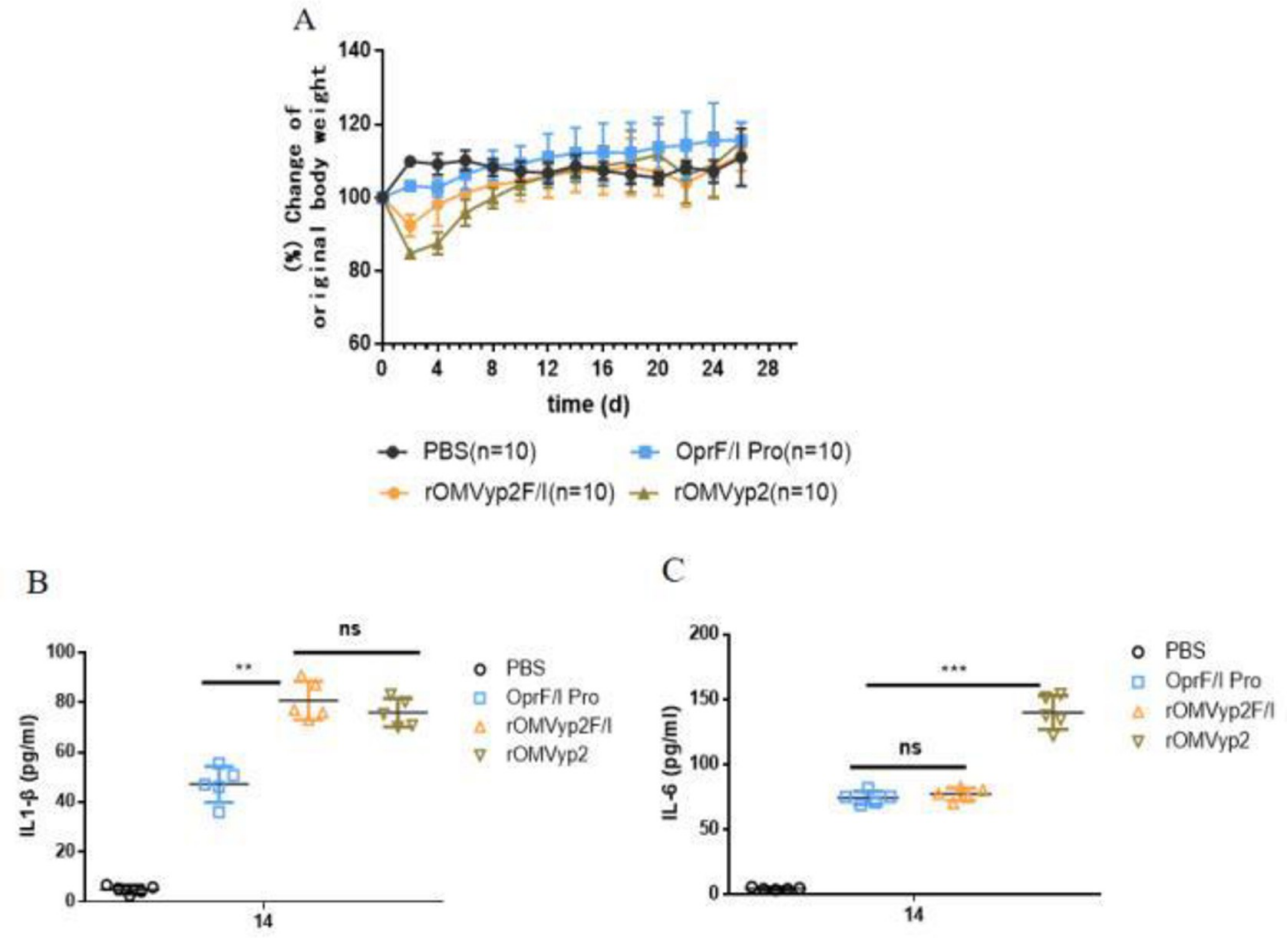
Changes of body weight and cytokines in rOMV_OprF/I_ group.

**Fig 5:**
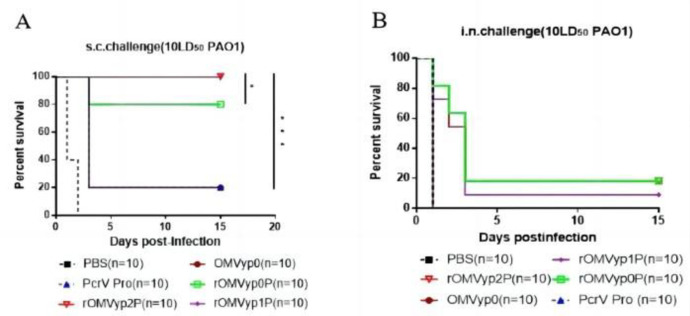
Immunoprotection in the rOMV_PcrV_ immune group.

Yp1Pand Yp2P gene deletion mutants were constructed by molecular biological methods **(S2 Fig 1 in S1 File)**, rOMVyp2P and rOMVyp2F/I were quantitatively identified by SDS-PAGE and Western Blot, a 31-kDa PcrV target protein and a 28-kDa OprF/I protein bands were obtained. This was consistent with the size of PcrV and OprF/I plasmids synthesized by Sangon Biotech company **(S2 Fig 2 and S2 Fig 3 in S1 File)**, indicating successful expression of the PcrV and OprF/I target protein in recombinant OMV. **(Fig 1 and S2 Fig 4 in S1 File**, and the relative content of the target proteins was analyzed by ImageJ semi-quantitative analysis through the grey value was about 6.5% and 3%. Subcellular localization results showed that most of the proteins in rOMVyp1P and rOMVyp2P were distributed in the cytoplasm, with a few localized in the cell membrane and the extracellular membrane. In contrast, most of the proteins in rOMVyp0P were distributed in the cell membrane, with a few found in the cytoplasm and the extracellular membrane **(S2 Fig 5 in S1 File).**

### 3.2 Transmission electron microscopy of recombinant OMVs morphology

**Fig 6:**
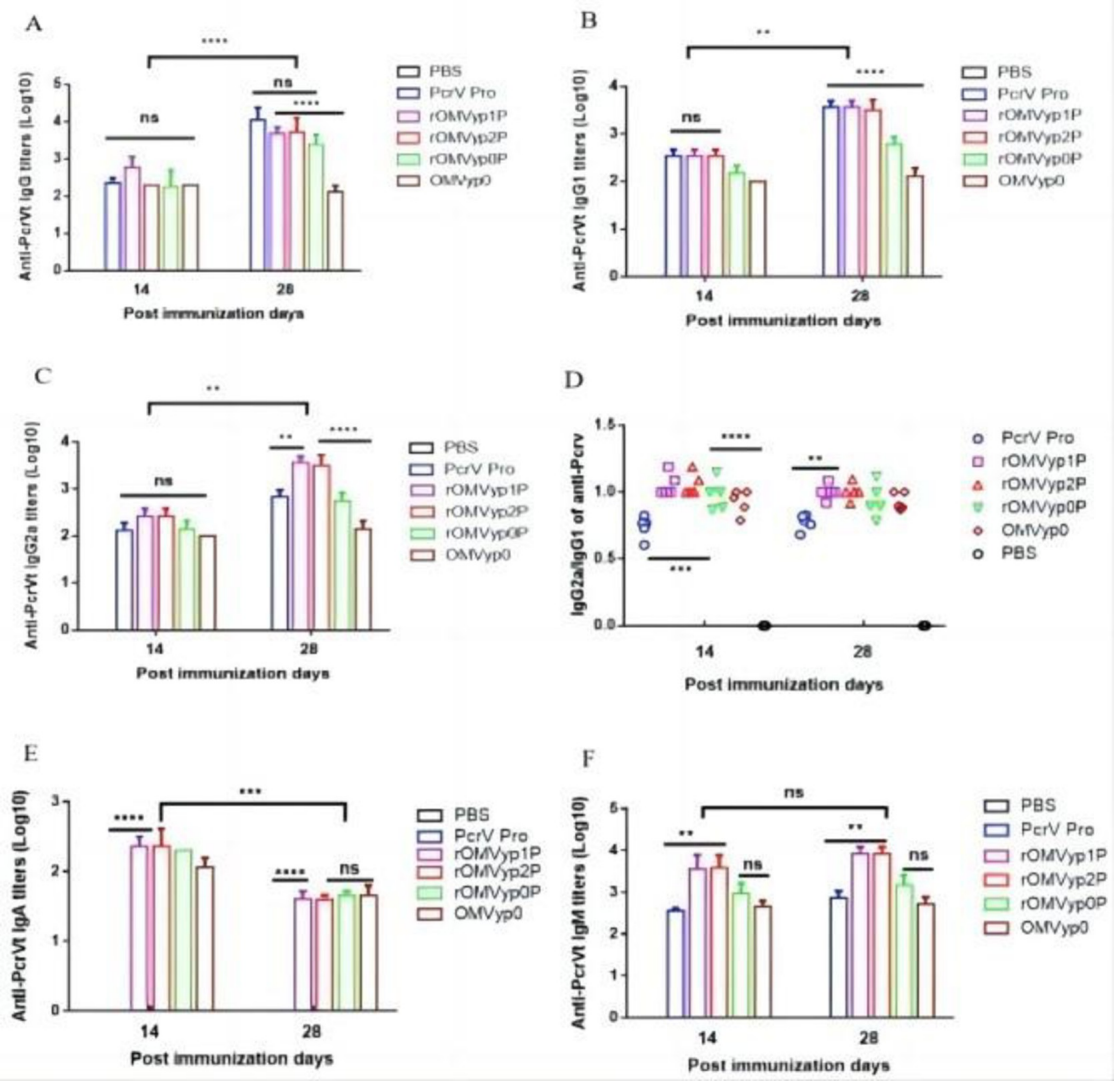
rOMV_PcrV_ immunome antibody level ELISA assay.

The diameter of rOMVs was found to be approximately 100–200 nm **(Fig 2)**, and the morphology of the three recombinant OMVs was with no big different.The particle size of rOMVyp0P was 118.9 ± 1.5 nm, and the number was 2.35e+10 ± 9.45e+08 particles/mL. rOMVyp1P had a particle size of 138.8 ± 1.3 nm and a number of 9.1e+09 ± 2.78e+08 particles/mL. rOMVyp2P had a particle size of 94.1 ± 2.0 nm and a number of 3.38e+10 ± 2.85e+09 particles/mL **(S2 Fig 6 in S1 File)**.

### 3.3 *LpxL* or *LpxA* gene deletion recombinant OMVs produced low toxic effects in mice

After immunization, we observed changes in the body weight of mice. We found that their body weight decreased significantly in the rOMVyp0P mice and gradually recovered after 4 d **(Fig 3A)**. We found that the serum inflammatory cytokines IL-6 differed significantly among the groups, whereas IL-1β did not differ significantly. rOMVyp1P and rOMVyp2P were significantly lower than the control group rOMVyp0P and OMVyp0**(Fig 3B and 3C)**, indicating that the toxicity of recombinant OMV was indeed reduced. The results of OprF/I group were similar to those of PcrV group **(Fig 4A–4C)**.

### 3.4 Effect of *LpxL* or *LpxA* gene deletion on PcrV immunoprotection against recombinant OMVs

After 42 d of immunization, we used two types of infection, subcutaneous induction of infection (called s.c.challenge) and nasal infection (called i.n.challenge). Each group received a subcutaneous induction of infection of the PAO1 strain at 10 LD50. The rOMVyp1P and rOMVyp2P groups provided 100% protection **(Fig 5A)**, and the PcrV protein group provided only 20% protection, which we analyzed may be due to the low amount of immunized PcrV protein, which further increased the amount of protein immunization. Each group was intra nasal infection with the PAO1 strain at 10 LD50, and the protective effects of rOMVyp1P, rOMVyp2P, and PcrV were observed. The results showed that all mice in the PcrV protein group died within one day, the rOMVyp1P group provided 10% protection, and the rOMVyp2P groups provided 20% protection **(Fig 5B)**.

The results showed that the differences in immune protection, we thought it might be caused by the different production levels of humoral immune antibodies, and then we tested the changes of antibody levels in each group.

### 3.5 Effect of *LpxL* or *LpxA* gene deletion on PcrV antibody levels against recombinant OMVs

Antibody level results showed IgG and IgM in the rOMVyp1P and rOMVyp2P groups were comparable to those in the rOMVyp0P group, while the levels of IgG2a and IgG1 antibodies were higher than those in the rOMVyp0P group. And we found that the IgG2a/IgG1 ratio of the rOMVyp1P and rOMVyp2P groups was approximately 1.05, which was significantly higher than that of the PcrV protein group, as observed in **(Fig 6)** rOMVyp1P and rOMVyp2P significantly increased the levels of various antibodies to PcrV and produced a more balanced Th1/Th2 immune response.

At the same time, we found that *LpxL* gene deletion not only reduced the toxicity of recombinant *Y*. *pseudotuberculosis* OMV and produced a high antibody level, but also kept a good protective effect against PAO1 pneumoniae infection, which was still up to 20%, which was significantly better than *LpxA* gene deletion recombinant *Y*. *pseudotuberculosis* OMV. Therefore, we selected OMV of recombinant *Y*. *pseudotuberculosis* that knocked out *LpxL* to conduct follow-up experiments on OprF/I immune response.

### 3.6 rOMV_OprF/I_ is capable of eliciting similar humoral immune responses, while concurrently generating identical subcutaneous immune protection

**Fig 7:**
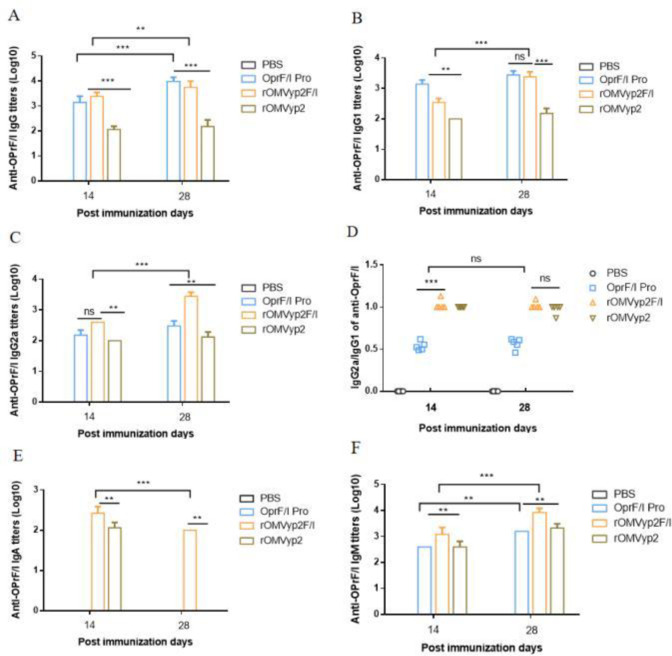
rOMV_OprF/I_ immunome antibody level ELISA assay.

At 14 and 28 days of ELISA detection, rOMVyp2F/I group also produced high levels of IgG, IgM and IgG1, which were higher than those of control. Additionally, the IgG2a/IgG1 ratio of the rOMVyp2F/I groups was approximately 1.12, which was significantly higher than that of the OprF/I protein group, indicating a Th1 and Th2 balanced immune response. The result shows that rOMVyp2F/I significantly increased the levels of various antibodies to OprF/I, which was consistent with the results of rOMVyp2P group **(Fig 7)**.

**Fig 8:**
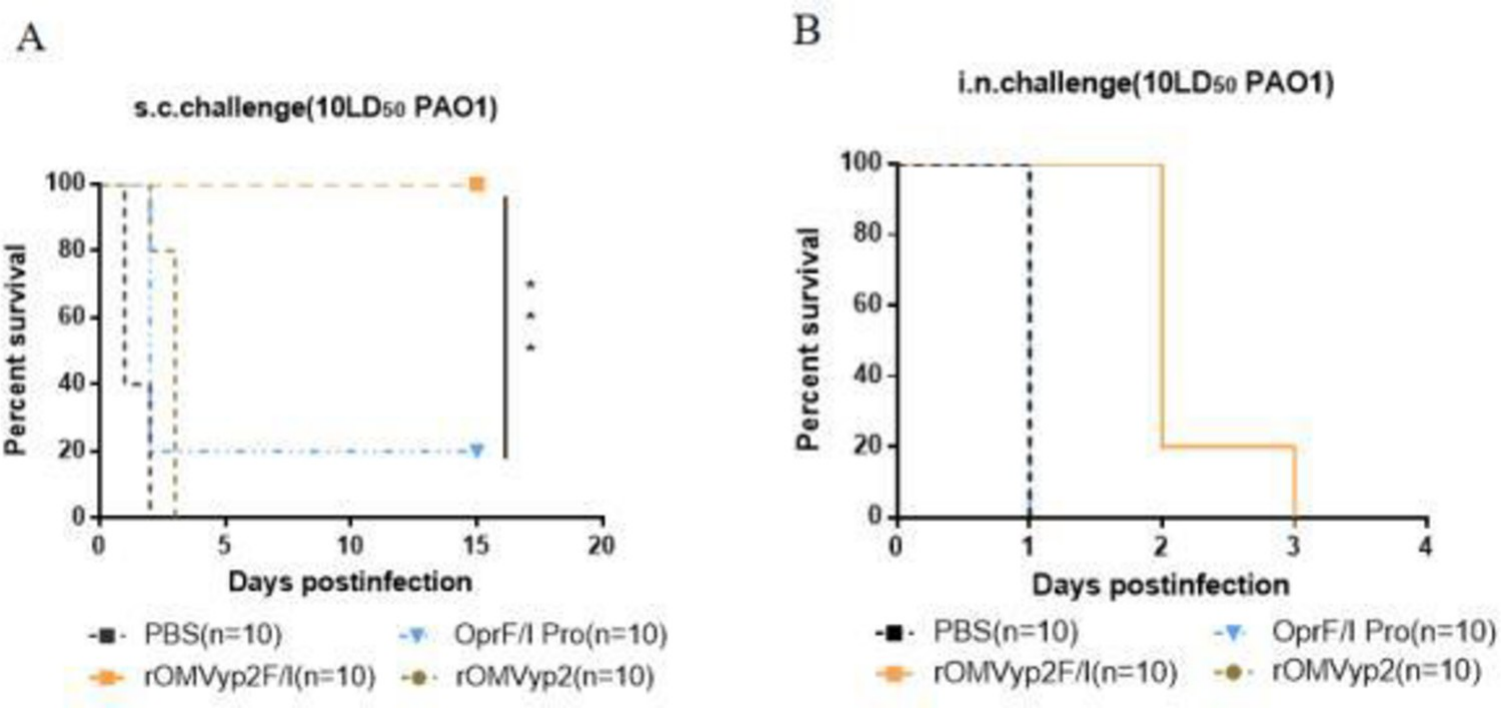
Immunoprotection in the rOMV_OprF/I_ immune group.

After 42 d of immunization, each group received a subcutaneous infection of the PAO1 strain at 10 LD50. The rOMVyp2F/I group provided 100% protection **(Fig 8A)**, and the OprF/I protein group also provided only 20% protection, we analyzed that this may be caused by the low immune dose of OprF/I protein, or the low expression level and poor expression effect of OprF/I protein. The next step is to increase the immune dose or protein amount. To better evaluate the immunoprotective effect of rOMVyp2F/I, we combined other forms of infection. At 42 d after inoculation, each group was intra nasal infection with the PAO1 strain at 10 LD50, and the protective effects of rOMVyp2F/I, rOMVyp2, and OprF/I were observed. The results showed that all mice in the OprF/I protein and rOMVyp2 groups died within 24h, the mice in the rOMVyp2F/I group died successively within three days, and no mice survived **(Fig 8B)**. This shows us that OMVs assists in eliciting similar humoral immune responses to different antigens, concurrently generating identical subcutaneous immune protection. However, there are differences in immune protection for the pulmonary region. The specific mechanism of action between OMV and antigen is still unclear, and further experiments are needed to explore and verify.

## 4. Discussion

The development of *P*. *aeruginosa* vaccines has always been the focus of research, and the selection and combination of antigens is the key to vaccine development **[32]**. Since *P*. *aeruginosa* contains a variety of antigens, there may be interactions between different antigens. In addition, different antigens may play a different role in vaccine research and development. **[33]**.

In this study, *Y*. *pseudotuberculosis* recombinant OMVs were used as a platform to validate the *P*. *aeruginosa* PcrV and OprF/I protein.

The average particle size of rOMVyp0P, rOMVyp1P and rOMVyp2P samples were found to be similar.By proteomic analysis of the protein composition of rOMVyp0P, rOMVyp1P, and rOMVyp2P, we identified the different proteins among the groups **(S2 Table 1 in S1 File)** and predicted the subcellular localization of these proteins, which may lay a theoretical basis for further study of the interaction between protein pathways **(S2 Fig 5 in S1 File).**We speculate that PcrV may interact with some proteins (Omp85, FetA, RmpM, etc.) or lipid analogs in rOMV, which leads to differences in charge number in rOMV, and does not exclude the influence of environmental factors. Specific reasons will be further explored in the follow-up topics. **[34]**, which can affect the OMVs differently. The *lpxL* or *lpxA* gene deletion might play the dominant role in proteomic differences **[35]**. Our analysis of KEGG results showed that rOMVyp2P significantly enhanced carbohydrate and energy metabolic pathways. The number of membrane transport and signaling genes increased significantly **(S2 Fig 7 in S1 File)**. In contrast, genes related to the immune system were not downregulated by modification. Based on literature analysis, we speculate that this may be one of the reasons why immunogenicity was maintained while antibody levels and immune protection were enhanced.It also laid a foundation for the development and mechanism of functional genes in the future **[36]**.

The IgG and IgM levels in rOMVyp2P group were slightly higher than those in rOMVyp1P group, at the same time, the immunoprotective evaluation showed that rOMVyp2P was significantly protective effect better than rOMVyp1P.Based on the above comprehensive analysis, we selected *ΔlpxL* rOMV_PcrV_ with better effect for the subsequent OprF/I antigen immune response experiment. However, it cannot be ruled out that PcrV and OprF/I proteins have different roles in recombinant OMV. The coupling between the proteins and their roles is not known, and whether they produce other immune-related chain reactions in mice needs to be studied in depth. At the same time, rOMVyp2F/I also produced high levels of IgG, IgM and IgG1 with the same trend as rOMVyp2 and similar antibody levels. It has been found that IgM provides the first line of defense during microbial infections prior to the production of IgG responses **[37]**. However, whether IgM is involved in this protection requires further investigation **[38,39]**. Compared with rOMVyp2F/I group, rOMVyp2P group showed more obvious immune protection. Mainly reflected in the intranasal PA01 challenge. We found that although PcrV and OprF/I are both important antigens of *P*. *aeruginosa*, the effects of OMV on different antigens are significantly different, which may be closely related to the interaction and rejection between OMV internal proteins and antigens, indicating that not all antigens are suitable to be used as antigen candidates for vaccine research. The specific reasons still need to be further explored and verified. Some studies have shown that antibody levels respond to immunoprotection, but after OMVs immunization this is not absolute; even no big difference in antibody levels, OMVs group still elicit high protection rates. This may be inextricably linked to our exploration of antigen suitability and selectivity, with a number of antigens remaining only as suitable as indicated and then used in practice for a variety of reasons **[40,41]**.

In this experiment, it was found that *Y*. *pseudotuberculosis* recombinant OMVs produced similar levels of antibodies against PcrV and OprF/I antigens, but there were differences in immune protection targeting lung infection. Based on whether it was only in *Y*. *pseudotuberculosis* or a common phenomenon, we also introduced *E*. *coli* OMVs to explore it. Since the antibody levels of the two antigens in OMVs are similar, but the immune protection in the lungs is different. We assumed that increasing the quantity of target antigens in OMVs can enhance immune protection. Therefore, we supplemented *E*. *coli* OMVs with quantitative mixing of the two antigens to support this. Similar to *Y*. *pseudotuberculosis*, the two antigens produce similar levels of antibodies. However, there was still a difference in immune protection, which was more pronounced in OprF/I, and the protective effect was worse **(S2 Fig 8 and S2 Fig 9 in S1 File)**. This further validates our hypothesis that OMVs exhibit differential immune protection against various antigens.

Through the analysis of EOMV-PcrV and EOMV-OprF/I, we reached a similar conclusion that the antibody levels of both are comparable and lower than those of rOMV. Although the antibody levels in the EOMV-OprF/I group were generally higher than in the OprF/I group, the immunoprotection was not as effective as expected.It is possible that OMVs affected the immunoprotection of OprF/I or that it may have been affected by the toxicity of *E*. *coli* OMVs, and the exact mechanism needs to be further investigated. *Y*. *pseudotuberculosis* rOMVyp2P and rOMVyp2F/I differed significantly in IgA antibody levels. IgA is associated with mucosal immunity, which it represents, and is also thought to play an important role in preventing *Salmonella* colonization in the thick mucus layer of the intestinal epithelium **[42,43]**. The two also differed markedly in their level of immunoprotection, with rOMVyp2P maintaining a 20% survival rate despite lung infection, compared no protection **(Figs 5B and 8B)**. rOMVyp2F/I elicited stronger TH1/TH2 type humoral immunity against OprF/I proteins than EOMV **(Fig 7, S2 Fig 9 in S1 File)**. OMVs had little effect on the antibody levels of different antigens, but immunoprotection against different antigens varied significantly. OMVs could increase the antibody levels of PcrV and OprF/I produce high immunoprotection rates at lower antibody levels. However, OMVs that delivered OprF/I produced significantly lower levels of immunoprotection, and the reasons for this need to be further explored **[25]**.

OMV contains LPS, which makes it somewhat toxic. How to reduce the toxicity of OMV has been an important topic of discussion. In this study, the difference of OMV toxicity in different groups can be reflected by the change of body weight of mice **[44]**; rOMVyp2P and rOMVyp2F/I were less toxic than control group, with smaller changes in body weight amplitude. Whether PcrV and OprF/I interacts with substances in recombinant OMVs, thereby attenuating their inflammatory properties, needs to be examined. IL-6 and IL-1β have also been used in inflammatory assays. The differences in IL-6 levels were not significant but could be seen to be different between the groups, whereas the values of IL-1β rOMVyp2P and rOMVyp2F/I were significantly lower than the control group. Knockouts of *LpxA* and *LpxL* have been proven to reduce toxicity **[1]**.

## 5. Conclusions

In this study, we found significant differences in the immune response of OMVs to different antigens. Recombinant *Y*. *pseudotuberculosis* OMVs constructed against PcrV and OprF/I antigens of *P*. *aeruginosa*. *ΔlpxL* rOMV_PcrV_ and *ΔlpxL* rOMV_OprF/I_ produce similar humoral immune responses and providing systemic immune protection, but their protection against *P*.*aeruginosa* PAO1 pulmonary infection is different. It was proved that OMVs had similar effects on different antigens, while there were some differences in pulmonary region. This lays the foundation for the selection of antigens for future vaccine development and an in-depth study of the mechanism of antigen action in OMVs.

## Supporting information

S1 Raw image (DOC)

S1 File (DOCX)
