# Correction: Effects of *Yersinia pseudotuberculosis* outer membrane vesicles on *Pseudomonas aeruginosa* antigens immune response

**DOI:** 10.1371/journal.pone.0321406

**Published:** 2025-03-28

**Authors:** Zhongxu Duan, Jingqi Song, Mingru Zhang, Zhe Zhang, Nan Li, Yuqin Fu, Zhe Sun, Tiancheng Lu, Siyuan Li, Mingyue Cao, Qingyu Wang, Chunhui Sun, Xiuran Wang

The funding statement for this paper is incorrect. The correct Funding statement is as follows: The financial support from the Jilin Provincial Natural Science Foundation of China (20220101335JC, 20210101404JC), The Youth Program of National Nature Science Foundation of China (82102786). Jilin Provincial Department of Education (JJKH20230377KJ).
