# Supplementary material for: Effects of Yersinia pseudotuberculosis outer membrane vesicles on Pseudomonas aeruginosa antigens immune response
Source: PLoS One. 2024 Dec 20;19(12):e0310652. doi: 10.1371/journal.pone.0310652 (PMC11661608; doi:10.1371/journal.pone.0310652)
Supplement: S1 File — (DOCX) [file pone.0310652.s002.docx]

S2_file

According to the upstream and downstream sequences of YP0 LpxA and LpxL genes of *Yersinia pseudotuberculosis*, primers were designed, according to *Pseudomonas aeruginosa* PcrV and OprF/I (E28-I294, Gene information source PAO1, Gene ID: 882997), the designed primer were sent to Shanghai Sheng gong Biological Engineering Co., Ltd. for synthesis.


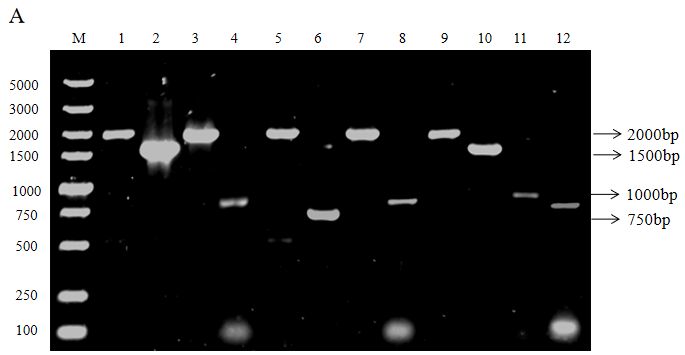


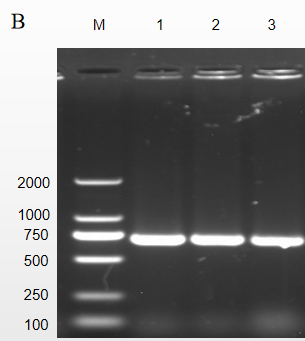


**S2 Fig 1 Identification of recombinant strains Yp0P (pNP214-PcrV), Yp1P (pNP214-PcrV), Yp2P (pNP214-PcrV) and Yp2F/I (pNP214-OprF/I)**

A.Recombinant pseudotuberculosis strains, primers LpxA, LpxL and PcrV 1-4：Yp0P (pNP214-PcrV), 5-8：Yp1P (pNP214-PcrV), 9-12: Yp2P (pNP214-PcrV);B. Recombinant pseudotuberculosis strains primers OprF/I 1-3: Yp2F/I (pNP214-OprF/I)


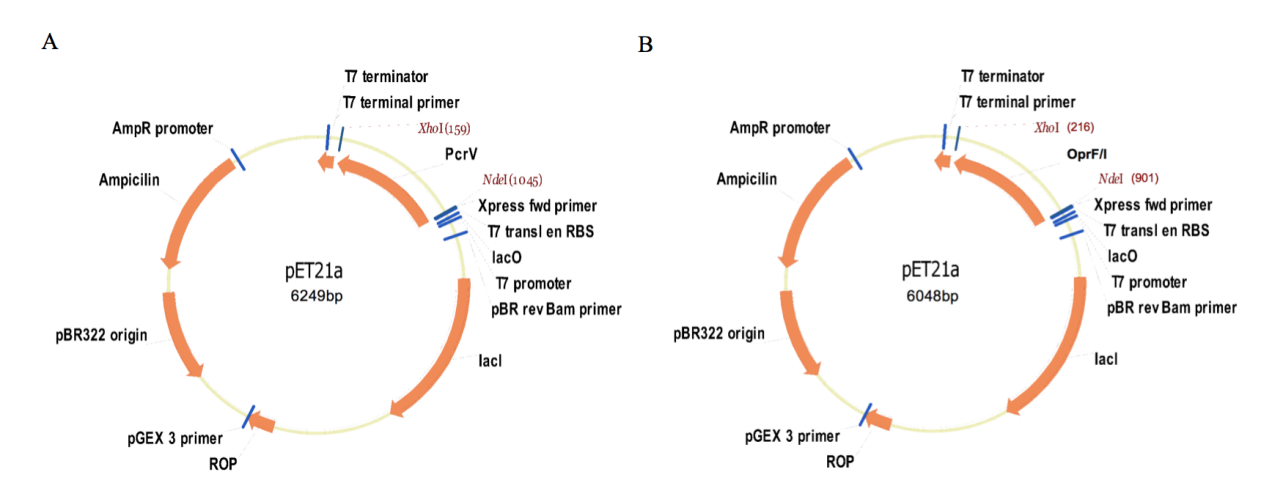


**S2 Fig 2** **Construction of pET21a-PcrV and pET21a-OprF/I expression vectors**

A. pET21a-PcrV; B. pET21a-OprF/I expression vector


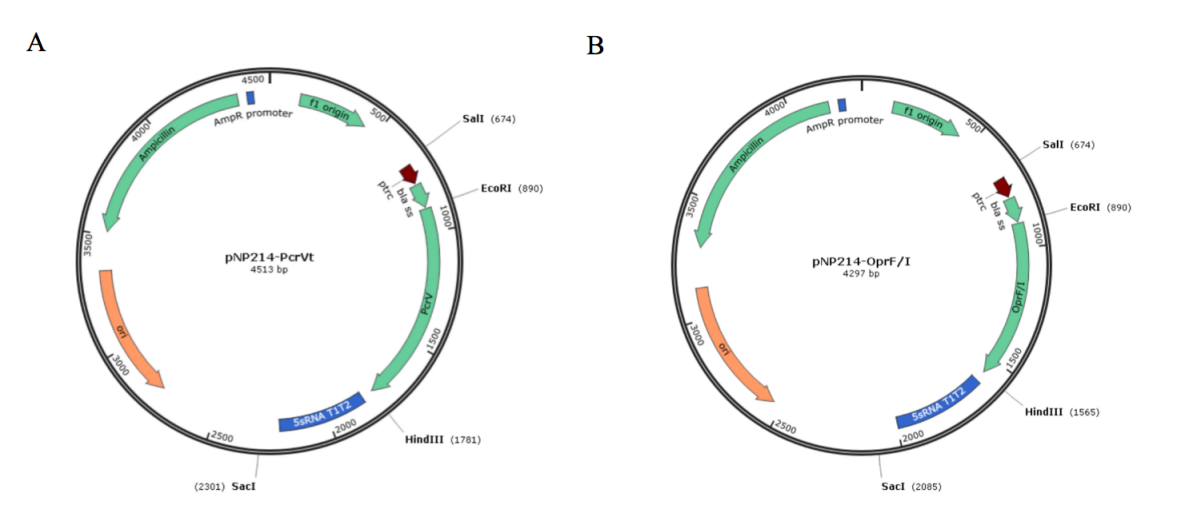


**S2 Fig 3**  **Design of pNP214 vector**

A.B. pNP214 expression vector.Based on pBluescript SK II(+), the ptrc promoter and 5ST1T2 terminator were used to construct an expression cassette, and the N-lead peptide of β-lactamase was used as the leader sequence (bla ss)


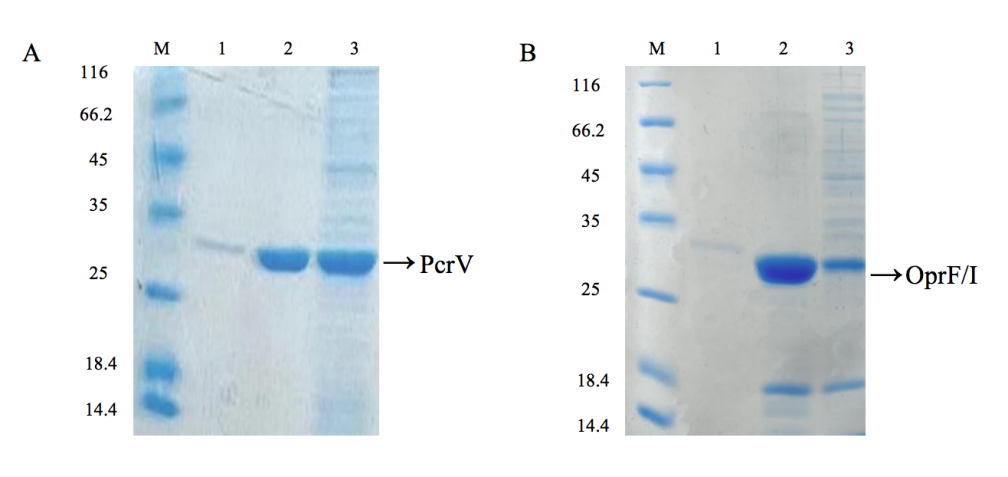


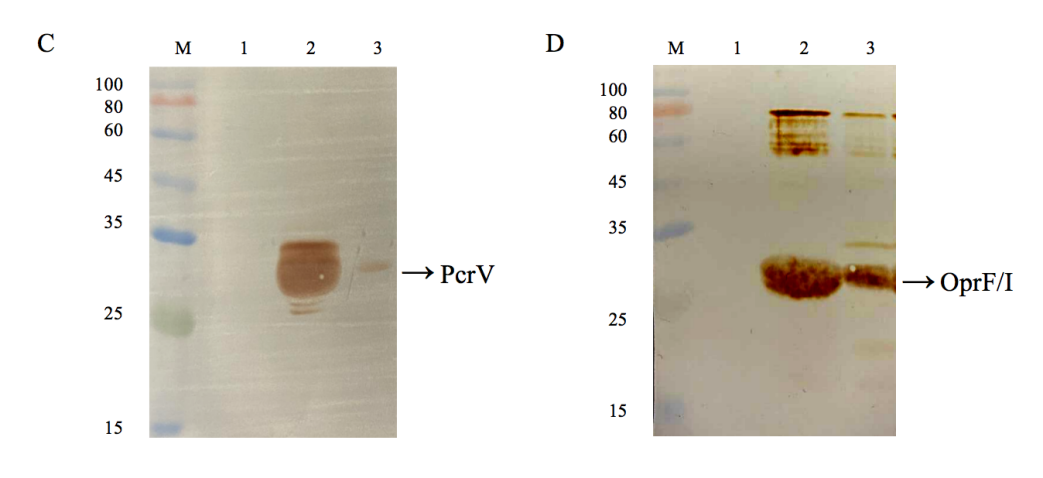


**S2 Fig 4** **Purification of recombinant proteins**

SDS-PAGE and Western blot analysis of recombinant protein A. PcrV SDS-PAGE: M.Protein molecular weight marker;1.negative control; 2.recombinant PcrV target protein; 3.positive control; B. OprF/I SDS-PAGE: M.Protein molecular weight marker;1.negative control; 2.recombinant OprF/I target protein; 3. positive control; C. PcrV western blot: M.Protein molecular weight marker;1.negative control; 2.recombinant PcrV target protein; 3.positive control; D. OprF/I western blot: M.Protein molecular weight marker;1.negative control; 2.recombinant OprF/I target protein; 3.positive control


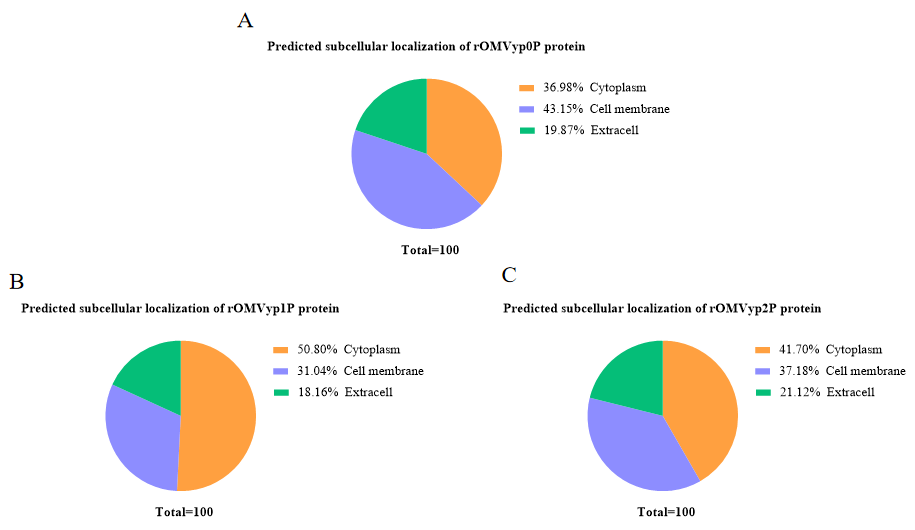


**S2 Fig 5** **Distribution of recombinant OMVs protein composition**

Predicted protein subcellular localization in rOMV，(A) rOMVyp0P;(B) rOMVyp1P;(C) rOMVyp2P.


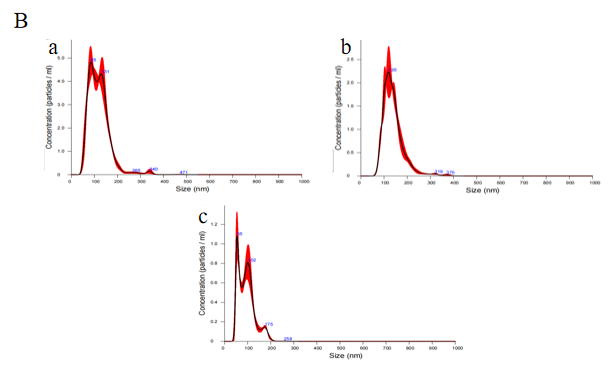


**S2 Fig 6 Determination of particle size and number of recombinant OMVs**

Determination of rOMV particle size and number, a-rOMVyp0P, b-rOMVyp1P, c-rOMVyp2P

We analyzed the protein sequences on the KEGG website and annotated their metabolic pathways with the following results.


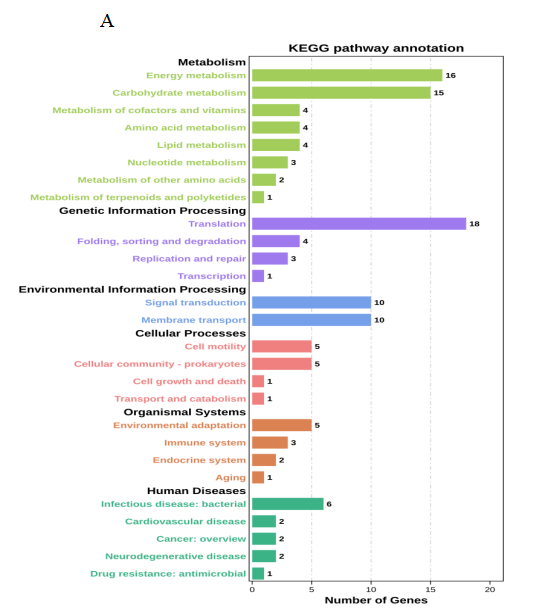


**S2 Fig 7** **rOMVs metabolome KEGG pathway annotation**

(A) rOMVyp0P; The metabolic pathway of rOMVyp0 protein was mainly concentrated in the translation region.


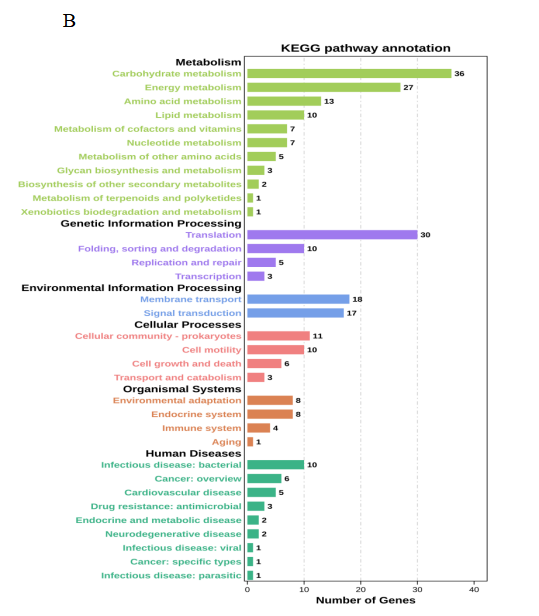


(B) rOMVyp1P; The protein metabolic pathway in rOMVyp1P is mainly focused on carbohydrate metabolism.


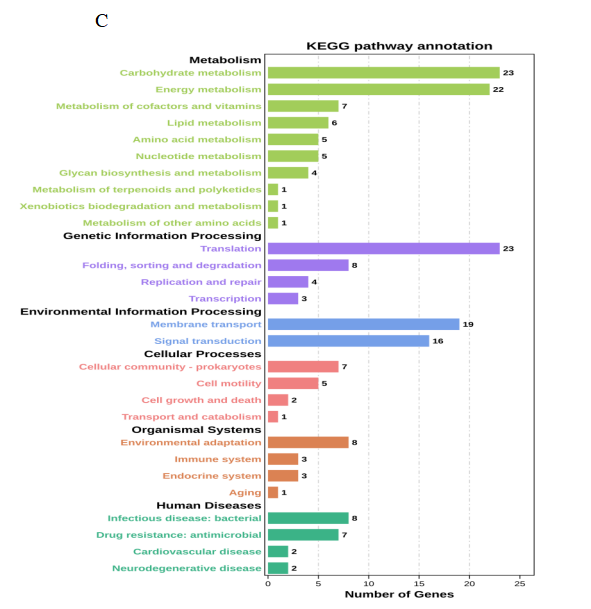


(C) rOMVyp2P; The protein metabolic pathways in rOMVyp2P mainly focus on carbohydrate metabolism, energy metabolism, membrane transport and signal transduction metabolism.

EOMV-PcrV: Each mouse was immunized 40 μg OMV+20 μg PcrV protein, and immunized twice. Because OMV is a self-adjuvant, the OMV solution can be mixed with an equal volume of 1/10×PBS solution. The dose of booster immunization is the same as that of primary immunization.


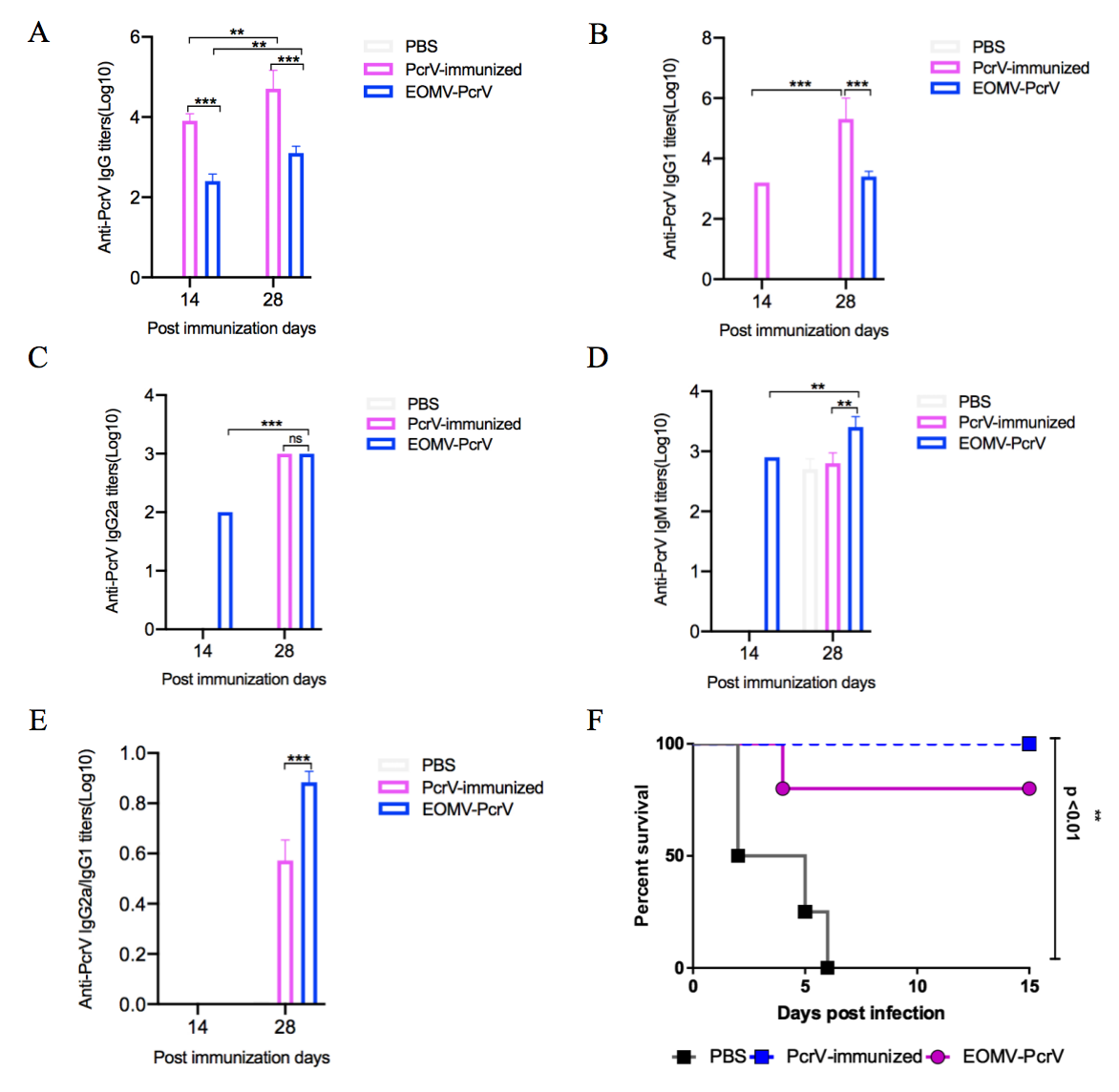


**S2 Fig 8** **Escherichia coli OMVs increase PcrV antibody levels but are highly toxic**

Elisa detection of antibody levels in EOMV-PcrV immune group and verification of immune protection

EOMV-OprF/I: Each mouse was immunized 40 μg OMV+20 μg OprF/I protein, and immunized twice. The OMV solution was mixed with an equal volume of 1/10×PBS solution, and the dose of booster immunization was the same as that of primary immunization.


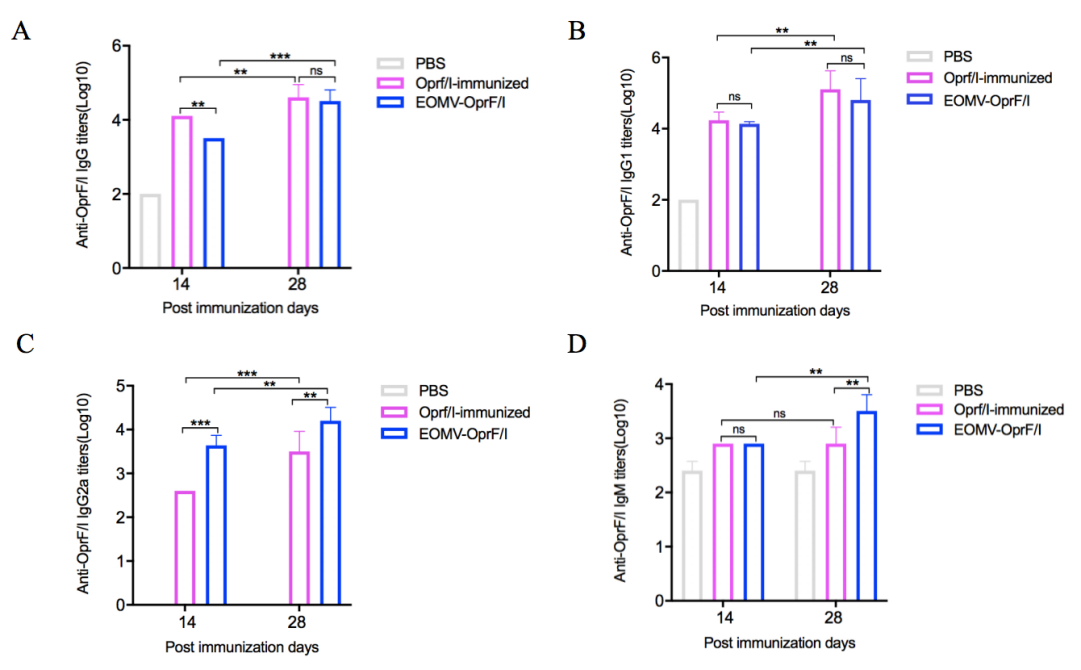


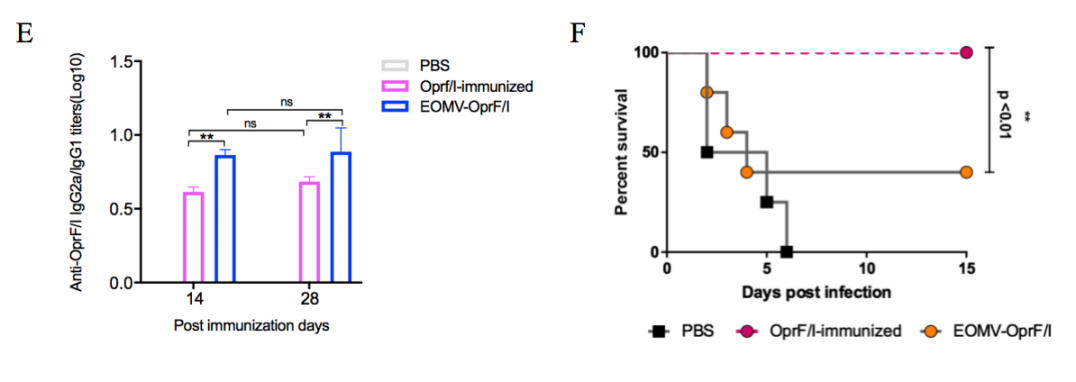


**S2 Fig 9** **Escherichia coli OMVs increase antibody levels to OprF/I but decreases immunoprotection**

Elisa detection of antibody levels in EOMV-OprF/I immune group and verification of immune protection

**S2 Table 1 rOMVyp0p、rOMVyp1p and rOMVyp2p differential proteins**

**Table 1-1** rOMVyp0p unique protein

| Protein name | Protein Information | Subcellular localization |
| --- | --- | --- |
| tr\|Q66BH6\|Q66BH6_YERPS | Recombination-associated protein RdgC OS=Yersinia pseudotuberculosis serotype I (strain IP32953) OX=273123 GN=rdgC PE=3 SV=1 | cytoplasm |
| tr\|A0A0E1NZ08\|A0A0E1NZ08_YERPA | HIT domain-containing protein OS=Yersinia pestis bv. Antiqua (strain Antiqua) OX=360102 GN=YPA_1911 PE=4 SV=1 | cytoplasm |
| tr\|A0A250ME27\|A0A250ME27_YERPU | Beta-lactamase OS=Yersinia pseudotuberculosis OX=633 PE=3 SV=1 | extracellular membrane |
| tr\|A0A0E1NWV1\|A0A0E1NWV1_YERPA | 30S ribosomal protein S1 OS=Yersinia pestis bv. Antiqua (strain Antiqua) OX=360102 GN=YPA_0683 PE=3 SV=1 | cytoplasm |
| tr\|A0A0E1NM93\|A0A0E1NM93_YERPA | Putative RNA-binding protein OS=Yersinia pestis bv. Antiqua (strain Antiqua) OX=360102 GN=YPA_2343 PE=4 SV=1 | cell membrane |
| tr\|A0A0U1QTN2\|A0A0U1QTN2_YERP3 | ATP-binding protein OS=Yersinia pseudotuberculosis serotype O:1b (strain IP 31758) OX=349747 GN=YpsIP31758_B0025 PE=4 SV=1 | cell membrane |
| tr\|A0A0E1NPV6\|A0A0E1NPV6_YERPA | Chemotaxis sensory transducer OS=Yersinia pestis bv. Antiqua (strain Antiqua) OX=360102 GN=YPA_3020 PE=4 SV=1 | cell membrane |
| tr\|A0A0E8XLT8\|A0A0E8XLT8_9GAMM | Anaerobic ribonucleoside triphosphate reductase OS=Yersinia wautersii OX=1341643 GN=nrdD PE=4 SV=1 | cytoplasm |
| tr\|A0A0E1NSF1\|A0A0E1NSF1_YERPA | 4-methyl-5(B-hydroxyethyl)-thiazol monophosphate biosynthesis enzyme OS=Yersinia pestis bv. Antiqua (strain Antiqua) OX=360102 GN=YPA_2666 PE=4 SV=1 | cytoplasm |
| tr\|A0A0E8XIJ5\|A0A0E8XIJ5_9GAMM | Membrane protein OS=Yersinia wautersii OX=1341643 GN=ERS008478_02117 PE=4 SV=1 | cell membrane |
| tr\|A0A380SBI7\|A0A380SBI7_YERPU | Bifunctional antitoxin/transcriptional repressor RelB OS=Yersinia pseudotuberculosis OX=633 GN=NCTC8580_04736 PE=3 SV=1 | cell membrane |
| tr\|A0A0E1NVY0\|A0A0E1NVY0_YERPA | Fimbrial usher protein OS=Yersinia pestis bv. Antiqua (strain Antiqua) OX=360102 GN=YPA_1298 PE=3 SV=1 | extracellular membrane |
| tr\|A0A0E8XIP0\|A0A0E8XIP0_9GAMM | Cell division topological specificity factor OS=Yersinia wautersii OX=1341643 GN=minE PE=3 SV=1 | cytoplasm |
| tr\|A0A0E1NT25\|A0A0E1NT25_YERPA | Putative carbon starvation protein A OS=Yersinia pestis bv. Antiqua (strain Antiqua) OX=360102 GN=YPA_1594 PE=3 SV=1 | cell membrane |
| tr\|A0A0T9NUN4\|A0A0T9NUN4_9GAMM | HAMP domain-containing protein OS=Yersinia similis OX=367190 GN=rpfG PE=4 SV=1 | cell membrane，cytoplasm |
| tr\|A0A0E8XLT6\|A0A0E8XLT6_9GAMM | Oligo-dipeptide/nickel ABC transporter substrate-binding protein OS=Yersinia wautersii OX=1341643 GN=dppA_3 PE=4 SV=1 | cell membrane |
| tr\|A0A2C8CUC7\|A0A2C8CUC7_YERPE | Flavin reductase OS=Yersinia pestis OX=632 GN=P2180_016 PE=4 SV=1 | cell membrane |
| tr\|A0A0E1NSX8\|A0A0E1NSX8_YERPA | ATP-dependent DNA helicase RecG OS=Yersinia pestis bv. Antiqua (strain Antiqua) OX=360102 GN=recG PE=3 SV=1 | cytoplasm |
| tr\|A0A0E1NX43\|A0A0E1NX43_YERPA | DNA helicase OS=Yersinia pestis bv. Antiqua (strain Antiqua) OX=360102 GN=YPA_0189 PE=3 SV=1 | cytoplasm |
| tr\|A0A0T9RMZ8\|A0A0T9RMZ8_9GAMM | Chaperone protein ClpB OS=Yersinia similis OX=367190 GN=clpB1 PE=3 SV=1 | cell membrane，cytoplasm |
| tr\|E8PS63\|E8PS63_YERPE | LuxR family protein OS=Yersinia pestis Java 9 OX=880632 GN=YPJ_pJARS359 PE=4 SV=1 | cell membrane，cytoplasm |
| tr\|A0A0E8XIU5\|A0A0E8XIU5_9GAMM | Type II secretion system protein E OS=Yersinia wautersii OX=1341643 GN=epsE PE=3 SV=1 | cell membrane |
| tr\|A0A0E1NVY2\|A0A0E1NVY2_YERPA | 3-isopropylmalate dehydrogenase OS=Yersinia pestis bv. Antiqua (strain Antiqua) OX=360102 GN=leuB PE=3 SV=1 | cytoplasm |
| tr\|A0A0E1NX29\|A0A0E1NX29_YERPA | Protein translocase subunit SecY OS=Yersinia pestis bv. Antiqua (strain Antiqua) OX=360102 GN=secY PE=3 SV=1 | cell membrane |
| tr\|A0A0T9R665\|A0A0T9R665_9GAMM | DNA-binding prophage protein OS=Yersinia similis OX=367190 GN=ERS008667_03477 PE=4 SV=1 | extracellular membrane |
| tr\|Q65AJ4\|Q65AJ4_YERPE | F1 capsule protein OS=Yersinia pestis OX=632 GN=caf1M PE=3 SV=1 | cell membrane |
| tr\|A0A3G5KDD2\|A0A3G5KDD2_YERPU | Phage protein OS=Yersinia pseudotuberculosis OX=633 GN=EGX52_08075 PE=4 SV=1 | cytoplasm |
| tr\|A0A0T9RKT0\|A0A0T9RKT0_9GAMM | Flagellar hook-length control protein OS=Yersinia similis OX=367190 GN=fliK2 PE=4 SV=1 | cell membrane |
| tr\|A0A0E1NWH9\|A0A0E1NWH9_YERPA | Isocitrate lyase OS=Yersinia pestis bv. Antiqua (strain Antiqua) OX=360102 GN=YPA_0018 PE=4 SV=1 | cytoplasm |
| tr\|Q666V4\|Q666V4_YERPS | Probable phage protein OS=Yersinia pseudotuberculosis serotype I (strain IP32953) OX=273123 GN=YPTB3142 PE=4 SV=1 | extracellular membrane |
| tr\|A0A0E1NWG6\|A0A0E1NWG6_YERPA | Putative kinase OS=Yersinia pestis bv. Antiqua (strain Antiqua) OX=360102 GN=YPA_0297 PE=4 SV=1 | extracellular membrane |
| tr\|A0A0E8XJJ7\|A0A0E8XJJ7_9GAMM | Helix-turn-helix， Fis-type OS=Yersinia wautersii OX=1341643 GN=zraR PE=4 SV=1 | cytoplasm |
| tr\|A0A0T9PS91\|A0A0T9PS91_9GAMM | Chromosome partition protein Smc OS=Yersinia similis OX=367190 GN=ERS008667_01541 PE=4 SV=1 | extracellular membrane |
| tr\|A0A0E8XGJ1\|A0A0E8XGJ1_9GAMM | Bifunctional glutamine synthetase adenylyltransferase/adenylyl-removing enzyme OS=Yersinia wautersii OX=1341643 GN=glnE PE=3 SV=1 | cytoplasm |
| tr\|A0A0E1NQF1\|A0A0E1NQF1_YERPA | Putative insulinase family protease OS=Yersinia pestis bv. Antiqua (strain Antiqua) OX=360102 GN=YPA_3819 PE=3 SV=1 | cytoplasm |
| tr\|Q66BH3\|Q66BH3_YERPS | Phage N-6-adenine-methyltransferase OS=Yersinia pseudotuberculosis serotype I (strain IP32953) OX=273123 GN=yfdM PE=4 SV=1 | extracellular membrane |
| tr\|A0A0E1NTY3\|A0A0E1NTY3_YERPA | Flagellar M-ring protein OS=Yersinia pestis bv. Antiqua (strain Antiqua) OX=360102 GN=YPA_1206 PE=3 SV=1 | cell membrane |
| tr\|A0A0E1NVL7\|A0A0E1NVL7_YERPA | Putative sugar transport ATP-binding protein OS=Yersinia pestis bv. Antiqua (strain Antiqua) OX=360102 GN=YPA_0306 PE=4 SV=1 | cell membrane |

**Table 1-2**:rOMVyp1unique protein

| Protein name | Protein Information | Subcellular localization |
| --- | --- | --- |
| tr\|A0A0E8XGP6\|A0A0E8XGP6_9GAMM | Enolase OS=Yersinia wautersii OX=1341643 GN=eno PE=3 SV=1 | cytoplasm，extracellular membrane |
| tr\|A0A0E8XNI8\|A0A0E8XNI8_9GAMM | Chaperonin GroEL OS=Yersinia wautersii OX=1341643 GN=groEL PE=3 SV=1 | cytoplasm |
| tr\|A0A0E1NZV3\|A0A0E1NZV3_YERPA | RNA polymerase sigma factor RpoD OS=Yersinia pestis bv. Antiqua (strain Antiqua) OX=360102 GN=rpoD PE=3 SV=1 | cell membrane，cytoplasm |
| tr\|A0A0E8XKM5\|A0A0E8XKM5_9GAMM | 30S ribosomal protein S10 OS=Yersinia wautersii OX=1341643 GN=nusE PE=3 SV=1 | cytoplasm |
| tr\|A0A0E1NRP6\|A0A0E1NRP6_YERPA | AAA_PrkA domain-containing protein OS=Yersinia pestis bv. Antiqua (strain Antiqua) OX=360102 GN=YPA_1511 PE=4 SV=1 | cytoplasm |
| tr\|A0A0E1NWX6\|A0A0E1NWX6_YERPA | Bifunctional protein PutA OS=Yersinia pestis bv. Antiqua (strain Antiqua) OX=360102 GN=YPA_1229 PE=3 SV=1 | cytoplasm |
| tr\|A0A0E8XSW3\|A0A0E8XSW3_9GAMM | Lysine--tRNA ligase OS=Yersinia wautersii OX=1341643 GN=lysS PE=3 SV=1 | cytoplasm |
| tr\|A0A0E8XKY1\|A0A0E8XKY1_9GAMM | 30S ribosomal protein S2 OS=Yersinia wautersii OX=1341643 GN=rpsB PE=3 SV=1 | cytoplasm |
| tr\|A0A0E8XKU9\|A0A0E8XKU9_9GAMM | 50S ribosomal protein L17 OS=Yersinia wautersii OX=1341643 GN=rplQ PE=3 SV=1 | cell membrane，cytoplasm |
| tr\|A0A0E8XIN1\|A0A0E8XIN1_9GAMM | Glucose-6-phosphate 1-dehydrogenase OS=Yersinia wautersii OX=1341643 GN=zwf PE=3 SV=1 | cytoplasm |
| tr\|A0A0E1NZB0\|A0A0E1NZB0_YERPA | Cell division protein FtsZ OS=Yersinia pestis bv. Antiqua (strain Antiqua) OX=360102 GN=ftsZ PE=3 SV=1 | cytoplasm |
| tr\|A0A0E1NXB6\|A0A0E1NXB6_YERPA | Aldehyde-alcohol dehydrogenase OS=Yersinia pestis bv. Antiqua (strain Antiqua) OX=360102 GN=YPA_1537 PE=3 SV=1 | cytoplasm |
| tr\|A0A0E1NS75\|A0A0E1NS75_YERPA | Cell shape-determining protein MreB OS=Yersinia pestis bv. Antiqua (strain Antiqua) OX=360102 GN=mreB PE=3 SV=1 | cytoplasm |
| tr\|A0A0E8XID6\|A0A0E8XID6_9GAMM | ATP-dependent RNA helicase RhlB OS=Yersinia wautersii OX=1341643 GN=rhlB PE=3 SV=1 | cytoplasm |
| tr\|A0A0E1NU45\|A0A0E1NU45_YERPA | Ribose-phosphate pyrophosphokinase OS=Yersinia pestis bv. Antiqua (strain Antiqua) OX=360102 GN=prs PE=3 SV=1 | cytoplasm |
| tr\|A0A0E1NT10\|A0A0E1NT10_YERPA | Formate dehydrogenase alpha subunit OS=Yersinia pestis bv. Antiqua (strain Antiqua) OX=360102 GN=YPA_3027 PE=3 SV=1 | cell membrane，cytoplasm |
| tr\|A0A0E1NMD9\|A0A0E1NMD9_YERPA | Cell division coordinator CpoB OS=Yersinia pestis bv. Antiqua (strain Antiqua) OX=360102 GN=cpoB PE=3 SV=1 | cell membrane，extracellular membrane |
| tr\|A0A0E1NMB3\|A0A0E1NMB3_YERPA | Ribonucleoside-diphosphate reductase OS=Yersinia pestis bv. Antiqua (strain Antiqua) OX=360102 GN=YPA_0928 PE=3 SV=1 | cytoplasm |
| sp\|Q0WBC9\|F16PA_YERPE | Fructose-1，6-bisphosphatase class 1 OS=Yersinia pestis OX=632 GN=fbp PE=3 SV=1 | cytoplasm |
| tr\|A0A0E8XNL3\|A0A0E8XNL3_9GAMM | Adenylosuccinate synthetase OS=Yersinia wautersii OX=1341643 GN=purA_2 PE=3 SV=1 | cytoplasm |
| tr\|A0A0E8XDB4\|A0A0E8XDB4_9GAMM | Serine-type D-Ala-D-Ala carboxypeptidase OS=Yersinia wautersii OX=1341643 GN=dacC PE=3 SV=1 | cell membrane |
| tr\|A0A0E8XK08\|A0A0E8XK08_9GAMM | Translation initiation factor IF-2 OS=Yersinia wautersii OX=1341643 GN=infB PE=3 SV=1 | cytoplasm |
| tr\|A0A0E1NY65\|A0A0E1NY65_YERPA | 50S ribosomal subunit assembly factor BipA OS=Yersinia pestis bv. Antiqua (strain Antiqua) OX=360102 GN=bipA PE=3 SV=1 | cytoplasm |
| tr\|A0A0E1NRT8\|A0A0E1NRT8_YERPA | Biotin carboxylase OS=Yersinia pestis bv. Antiqua (strain Antiqua) OX=360102 GN=YPA_3671 PE=4 SV=1 | cell membrane |
| tr\|A0A0E1NXP2\|A0A0E1NXP2_YERPA | Pyruvate kinase OS=Yersinia pestis bv. Antiqua (strain Antiqua) OX=360102 GN=YPA_1447 PE=3 SV=1 | cytoplasm |
| tr\|A0A0E8XNA2\|A0A0E8XNA2_9GAMM | ATP-dependent 6-phosphofructokinase OS=Yersinia wautersii OX=1341643 GN=pfkA PE=3 SV=1 | cytoplasm |
| tr\|A0A0E8XSK3\|A0A0E8XSK3_9GAMM | Succinate--CoA ligase [ADP-forming] subunit beta OS=Yersinia wautersii OX=1341643 GN=sucC PE=3 SV=1 | cell membrane，cytoplasm |
| tr\|A0A0E1NUK6\|A0A0E1NUK6_YERPA | NAD(P) transhydrogenase subunit alpha OS=Yersinia pestis bv. Antiqua (strain Antiqua) OX=360102 GN=YPA_1652 PE=3 SV=1 | cytoplasm |
| tr\|A0A0E1NVT3\|A0A0E1NVT3_YERPA | Succinate--CoA ligase [ADP-forming] subunit alpha OS=Yersinia pestis bv. Antiqua (strain Antiqua) OX=360102 GN=sucD PE=3 SV=1 | cytoplasm |
| tr\|A0A0E8XG91\|A0A0E8XG91_9GAMM | Bifunctional polymyxin resistance protein ArnA OS=Yersinia wautersii OX=1341643 GN=arnA PE=3 SV=1 | cytoplasm |
| tr\|A0A0E8XHD3\|A0A0E8XHD3_9GAMM | ATP-dependent Clp protease ATP-binding subunit ClpX OS=Yersinia wautersii OX=1341643 GN=clpX PE=3 SV=1 | cell membrane |
| tr\|A0A0E8XKP7\|A0A0E8XKP7_9GAMM | 50S ribosomal protein L15 OS=Yersinia wautersii OX=1341643 GN=rplO PE=3 SV=1 | cytoplasm |
| tr\|A0A0E1NLK5\|A0A0E1NLK5_YERPA | 3-oxoacyl-[acyl-carrier-protein] synthase I OS=Yersinia pestis bv. Antiqua (strain Antiqua) OX=360102 GN=YPA_2086 PE=3 SV=1 | extracellular membrane |
| tr\|A0A0E1NR38\|A0A0E1NR38_YERPA | Thioredoxin reductase OS=Yersinia pestis bv. Antiqua (strain Antiqua) OX=360102 GN=YPA_0664 PE=3 SV=1 | cytoplasm |
| tr\|A0A0E1NT27\|A0A0E1NT27_YERPA | Aspartate ammonia-lyase OS=Yersinia pestis bv. Antiqua (strain Antiqua) OX=360102 GN=YPA_3934 PE=3 SV=1 | cytoplasm |
| tr\|A0A0E1NXC4\|A0A0E1NXC4_YERPA | Flagellar hook protein FlgE OS=Yersinia pestis bv. Antiqua (strain Antiqua) OX=360102 GN=YPA_1177 PE=3 SV=1 | extracellular membrane |
| tr\|A0A0E8XJF0\|A0A0E8XJF0_9GAMM | 30S ribosomal protein S18 OS=Yersinia wautersii OX=1341643 GN=rpsR PE=3 SV=1 | cell membrane |
| tr\|A0A0E1NXW5\|A0A0E1NXW5_YERPA | Phospholipid-binding protein MlaC OS=Yersinia pestis bv. Antiqua (strain Antiqua) OX=360102 GN=YPA_3731 PE=4 SV=1 | extracellular membrane |
| tr\|A0A0E8XKA0\|A0A0E8XKA0_9GAMM | 50S ribosomal protein L16 OS=Yersinia wautersii OX=1341643 GN=rplP PE=3 SV=1 | cytoplasm |
| tr\|A0A0E1NS12\|A0A0E1NS12_YERPA | Aconitate hydratase OS=Yersinia pestis bv. Antiqua (strain Antiqua) OX=360102 GN=YPA_1580 PE=3 SV=1 | cytoplasm |
| tr\|A0A0E8XFL7\|A0A0E8XFL7_9GAMM | Chaperone protein HtpG OS=Yersinia wautersii OX=1341643 GN=htpG PE=3 SV=1 | cell membrane，cytoplasm |
| tr\|A0A0E8XQB0\|A0A0E8XQB0_9GAMM | Ribonuclease PH OS=Yersinia wautersii OX=1341643 GN=rph PE=3 SV=1 | cytoplasm |
| tr\|A0A0E1NLZ7\|A0A0E1NLZ7_YERPA | Phosphoenolpyruvate synthase OS=Yersinia pestis bv. Antiqua (strain Antiqua) OX=360102 GN=YPA_1753 PE=3 SV=1 | cytoplasm |
| tr\|A0A0E8XHT6\|A0A0E8XHT6_9GAMM | Uracil phosphoribosyltransferase OS=Yersinia wautersii OX=1341643 GN=upp PE=3 SV=1 | cytoplasm |
| tr\|A0A0E8XIY2\|A0A0E8XIY2_9GAMM | Aspartate--tRNA ligase OS=Yersinia wautersii OX=1341643 GN=aspS PE=3 SV=1 | cytoplasm |
| tr\|A0A0E1NXJ1\|A0A0E1NXJ1_YERPA | Methyl-accepting chemotaxis protein OS=Yersinia pestis bv. Antiqua (strain Antiqua) OX=360102 GN=YPA_1845 PE=3 SV=1 | cell membrane |
| tr\|A0A0E8XQ61\|A0A0E8XQ61_9GAMM | ATP-dependent protease ATPase subunit HslU OS=Yersinia wautersii OX=1341643 GN=hslU PE=3 SV=1 | cell membrane，cytoplasm |
| tr\|A0A0E1NNE3\|A0A0E1NNE3_YERPA | ATP-dependent RNA helicase DbpA OS=Yersinia pestis bv. Antiqua (strain Antiqua) OX=360102 GN=dbpA PE=3 SV=1 | cell membrane，cytoplasm |
| tr\|A0A0E8XR21\|A0A0E8XR21_9GAMM | Major capsid protein OS=Yersinia wautersii OX=1341643 GN=ERS008478_04136 PE=4 SV=1 | extracellular membrane |
| tr\|A0A0E1NYZ5\|A0A0E1NYZ5_YERPA | Lipopolysaccharide assembly protein B OS=Yersinia pestis bv. Antiqua (strain Antiqua) OX=360102 GN=lapB PE=3 SV=1 | cytoplasm |
| tr\|A0A0E1NXH1\|A0A0E1NXH1_YERPA | Oxoglutarate dehydrogenase (succinyl-transferring) OS=Yersinia pestis bv. Antiqua (strain Antiqua) OX=360102 GN=YPA_0591 PE=3 SV=1 | cytoplasm |
| tr\|A0A0E8XEA4\|A0A0E8XEA4_9GAMM | CTP synthase OS=Yersinia wautersii OX=1341643 GN=pyrG PE=3 SV=1 | cytoplasm |
| tr\|A0A250MG33\|A0A250MG33_YERPU | Tn3 transposase OS=Yersinia pseudotuberculosis OX=633 PE=3 SV=1 | cytoplasm，extracellular membrane |
| tr\|A0A0E1NU24\|A0A0E1NU24_YERPA | VacJ lipoprotein OS=Yersinia pestis bv. Antiqua (strain Antiqua) OX=360102 GN=YPA_2101 PE=3 SV=1 | extracellular membrane |
| tr\|A0A2C8CVI3\|A0A2C8CVI3_YERPE | Site-specific DNA-methyltransferase (adenine-specific) OS=Yersinia pestis OX=632 GN=dam PE=3 SV=1 | extracellular membrane |
| tr\|A0A0E1NN00\|A0A0E1NN00_YERPA | Tellurium resistance protein OS=Yersinia pestis bv. Antiqua (strain Antiqua) OX=360102 GN=YPA_3984 PE=3 SV=1 | cell membrane |
| tr\|A0A0E1NWM2\|A0A0E1NWM2_YERPA | Putative type III secretion apparatus protein OS=Yersinia pestis bv. Antiqua (strain Antiqua) OX=360102 GN=YPA_4010 PE=3 SV=1 | cell membrane |
| tr\|A0A0E8XKI9\|A0A0E8XKI9_9GAMM | Branched-chain amino acid-binding protein OS=Yersinia wautersii OX=1341643 GN=livK PE=3 SV=1 | cytoplasm |
| tr\|A0A0E8XGZ9\|A0A0E8XGZ9_9GAMM | Flagellar biosynthesis protein FlhA OS=Yersinia wautersii OX=1341643 GN=flhA_1 PE=3 SV=1 | cell membrane |
| tr\|A0A8K1DSG2\|A0A8K1DSG2_9CAUD | Phage protein OS=Yersinia phage PYps55T OX=2764921 GN=PYps55T_175 PE=4 SV=1 | cell membrane，cytoplasm，extracellular membrane |
| tr\|A0A0E8XM25\|A0A0E8XM25_9GAMM | S-adenosylmethionine synthase OS=Yersinia wautersii OX=1341643 GN=metK PE=3 SV=1 | cytoplasm |
| tr\|A0A0E8XGU9\|A0A0E8XGU9_9GAMM | Chaperone protein Skp OS=Yersinia wautersii OX=1341643 GN=hlpA PE=3 SV=1 | cytoplasm |
| tr\|A0A0E1NTT7\|A0A0E1NTT7_YERPA | Periplasmic pectate lyase OS=Yersinia pestis bv. Antiqua (strain Antiqua) OX=360102 GN=YPA_3822 PE=4 SV=1 | cytoplasm |
| tr\|A0A0E1NW09\|A0A0E1NW09_YERPA | 2-amino-3-ketobutyrate coenzyme A ligase OS=Yersinia pestis bv. Antiqua (strain Antiqua) OX=360102 GN=kbl PE=3 SV=1 | cytoplasm |
| tr\|A0A0E1NMY9\|A0A0E1NMY9_YERPA | Yop targeting protein OS=Yersinia pestis bv. Antiqua (strain Antiqua) OX=360102 GN=YPA_CD0032 PE=4 SV=1 | cell membrane |
| tr\|A0A0E1NRS4\|A0A0E1NRS4_YERPA | Type VI secretion system membrane subunit TssM OS=Yersinia pestis bv. Antiqua (strain Antiqua) OX=360102 GN=YPA_3406 PE=4 SV=1 | cell membrane |
| tr\|A0A0E1NUA2\|A0A0E1NUA2_YERPA | Putative AMP-binding enzyme-family protein OS=Yersinia pestis bv. Antiqua (strain Antiqua) OX=360102 GN=YPA_3561 PE=4 SV=1 | cytoplasm |
| tr\|A0A0E8XJB3\|A0A0E8XJB3_9GAMM | Isochorismate synthase MenF OS=Yersinia wautersii OX=1341643 GN=menF PE=3 SV=1 | cytoplasm |
| tr\|A0A0T9RC08\|A0A0T9RC08_9GAMM | Integrase family protein OS=Yersinia similis OX=367190 GN=ERS008667_03694 PE=4 SV=1 | cytoplasm |
| tr\|A0A3G5KNP0\|A0A3G5KNP0_YERPU | 30S ribosomal protein S15 OS=Yersinia pseudotuberculosis OX=633 GN=rpsO PE=3 SV=1 | cytoplasm |
| tr\|A0A0T9NZK8\|A0A0T9NZK8_9GAMM | Variant surface antigen rifin OS=Yersinia similis OX=367190 GN=ERS008667_00452 PE=4 SV=1 | extracellular membrane |
| tr\|A0A0E1P1S6\|A0A0E1P1S6_YERPA | Putative flagellar assembly protein OS=Yersinia pestis bv. Antiqua (strain Antiqua) OX=360102 GN=YPA_3073 PE=4 SV=1 | cytoplasm |
| tr\|A0A250MEF1\|A0A250MEF1_YERPU | TraE OS=Yersinia pseudotuberculosis OX=633 PE=4 SV=1 | cell membrane |
| tr\|A0A0E8XKF9\|A0A0E8XKF9_9GAMM | Glycerol-3-phosphate acyltransferase OS=Yersinia wautersii OX=1341643 GN=pslB PE=3 SV=1 | cell membrane |
| tr\|A0A0E1NZH5\|A0A0E1NZH5_YERPA | ATP-dependent Clp protease ATP-binding subunit ClpA OS=Yersinia pestis bv. Antiqua (strain Antiqua) OX=360102 GN=YPA_0658 PE=3 SV=1 | cell membrane，cytoplasm |
| tr\|A0A0E8XMR4\|A0A0E8XMR4_9GAMM | ABC transporter ATP-binding protein OS=Yersinia wautersii OX=1341643 GN=potA_2 PE=4 SV=1 | cell membrane |
| tr\|A0A0E1NX41\|A0A0E1NX41_YERPA | Na(+)-translocating NADH-quinone reductase subunit B OS=Yersinia pestis bv. Antiqua (strain Antiqua) OX=360102 GN=nqrB PE=3 SV=1 | cell membrane |
| tr\|A0A0E8XPC8\|A0A0E8XPC8_9GAMM | Glucose-1-phosphate adenylyltransferase OS=Yersinia wautersii OX=1341643 GN=glgC PE=3 SV=1 | cytoplasm |
| tr\|A0A0E1NR61\|A0A0E1NR61_YERPA | 3-oxoacyl-[acyl-carrier-protein] synthase 2 OS=Yersinia pestis bv. Antiqua (strain Antiqua) OX=360102 GN=YPA_1924 PE=3 SV=1 | extracellular membrane |
| tr\|A0A0T9RAJ2\|A0A0T9RAJ2_9GAMM | Aminotransferase OS=Yersinia similis OX=367190 GN=tyrB PE=3 SV=1 | cytoplasm |
| tr\|A0A0E1NLL1\|A0A0E1NLL1_YERPA | DNA translocase FtsK OS=Yersinia pestis bv. Antiqua (strain Antiqua) OX=360102 GN=YPA_0666 PE=3 SV=1 | cell membrane |
| tr\|A0A0E8XLQ4\|A0A0E8XLQ4_9GAMM | Multidrug resistance protein MdtA OS=Yersinia wautersii OX=1341643 GN=mdtE PE=3 SV=1 | cell membrane |
| tr\|A0A0E8XJ75\|A0A0E8XJ75_9GAMM | Acetate kinase OS=Yersinia wautersii OX=1341643 GN=ack PE=3 SV=1 | cytoplasm |
| tr\|A0A0E8XMZ2\|A0A0E8XMZ2_9GAMM | Glycine--tRNA ligase alpha subunit OS=Yersinia wautersii OX=1341643 GN=glyQ PE=3 SV=1 | cytoplasm |
| tr\|A0A0T9MHH8\|A0A0T9MHH8_YERPU | PAAR domain-containing protein OS=Yersinia pseudotuberculosis OX=633 GN=NCTC8580_00592 PE=4 SV=1 | extracellular membrane |
| tr\|A0A0E1NRA8\|A0A0E1NRA8_YERPA | Spheroplast protein Y OS=Yersinia pestis bv. Antiqua (strain Antiqua) OX=360102 GN=YPA_3468 PE=3 SV=1 | cytoplasm |
| tr\|A0A0E1NML2\|A0A0E1NML2_YERPA | ATP-dependent RNA helicase SrmB OS=Yersinia pestis bv. Antiqua (strain Antiqua) OX=360102 GN=srmB PE=3 SV=1 | cytoplasm |
| tr\|A0A0U1QTB4\|A0A0U1QTB4_YERP3 | HTH cro/C1-type domain-containing protein OS=Yersinia pseudotuberculosis serotype O:1b (strain IP 31758) OX=349747 GN=YpsIP31758_A0035 PE=4 SV=1 | cell membrane |
| tr\|A0A0T9J733\|A0A0T9J733_YERPU | AP endonuclease OS=Yersinia pseudotuberculosis OX=633 GN=NCTC8580_02418 PE=4 SV=1 | cytoplasm |
| tr\|A0A0E8XDU5\|A0A0E8XDU5_9GAMM | Elongation factor 4 OS=Yersinia wautersii OX=1341643 GN=lepA PE=3 SV=1 | cytoplasm |
| tr\|A0A0E1NQ73\|A0A0E1NQ73_YERPA | 4-hydroxyphenylacetate 3-monooxygenase OS=Yersinia pestis bv. Antiqua (strain Antiqua) OX=360102 GN=YPA_1141 PE=4 SV=1 | cytoplasm |
| tr\|A0A0E1NYG9\|A0A0E1NYG9_YERPA | S1 motif domain-containing protein OS=Yersinia pestis bv. Antiqua (strain Antiqua) OX=360102 GN=YPA_3335 PE=4 SV=1 | cytoplasm |
| tr\|Q65AG1\|Q65AG1_YERPE | TraI protein OS=Yersinia pestis OX=632 GN=traI PE=4 SV=1 | cytoplasm |
| tr\|A0A0E1NT41\|A0A0E1NT41_YERPA | Putative lipoprotein OS=Yersinia pestis bv. Antiqua (strain Antiqua) OX=360102 GN=YPA_2827 PE=4 SV=1 | extracellular membrane |
| tr\|A0A0E8XLD1\|A0A0E8XLD1_9GAMM | Gluconate utilization system Gnt-I transcriptional repressor OS=Yersinia wautersii OX=1341643 GN=gntR_2 PE=4 SV=1 | cytoplasm |
| tr\|A0A0E8XKP2\|A0A0E8XKP2_9GAMM | 30S ribosomal protein S8 OS=Yersinia wautersii OX=1341643 GN=rpsH PE=3 SV=1 | cytoplasm |
| tr\|A0A0E1NP60\|A0A0E1NP60_YERPA | Putative yfeABCD locus regulator OS=Yersinia pestis bv. Antiqua (strain Antiqua) OX=360102 GN=YPA_1789 PE=4 SV=1 | cell membrane |
| tr\|A0A0E8XRD1\|A0A0E8XRD1_9GAMM | Protein ViaA OS=Yersinia wautersii OX=1341643 GN=viaA PE=3 SV=1 | cytoplasm |
| tr\|A0A0T9QJ93\|A0A0T9QJ93_9GAMM | Putative outer membrane usher protein OS=Yersinia similis OX=367190 GN=htrE_1 PE=3 SV=1 | extracellular membrane |
| tr\|A0A0E8XMB5\|A0A0E8XMB5_9GAMM | Major capsid protein OS=Yersinia wautersii OX=1341643 GN=ERS008478_02809 PE=4 SV=1 | extracellular membrane |
| tr\|A0A250MEB7\|A0A250MEB7_YERPU | LigA OS=Yersinia pseudotuberculosis OX=633 PE=4 SV=1 | extracellular membrane |
| tr\|A0A0E8XNC6\|A0A0E8XNC6_9GAMM | 50S ribosomal protein L11 OS=Yersinia wautersii OX=1341643 GN=rplK PE=3 SV=1 | cell membrane，cytoplasm |
| tr\|A0A0E1NVY3\|A0A0E1NVY3_YERPA | Type VI secretion system baseplate subunit TssF OS=Yersinia pestis bv. Antiqua (strain Antiqua) OX=360102 GN=YPA_3396 PE=4 SV=1 | cell membrane |
| tr\|A0A0E1NVU3\|A0A0E1NVU3_YERPA | AMP nucleosidase OS=Yersinia pestis bv. Antiqua (strain Antiqua) OX=360102 GN=YPA_0507 PE=4 SV=1 | cell membrane，cytoplasm |
| tr\|A0A0E8XFY9\|A0A0E8XFY9_9GAMM | 3-phosphoshikimate 1-carboxyvinyltransferase OS=Yersinia wautersii OX=1341643 GN=aroA PE=3 SV=1 | cytoplasm |
| tr\|A0A0E1NVQ1\|A0A0E1NVQ1_YERPA | Nucleoside permease OS=Yersinia pestis bv. Antiqua (strain Antiqua) OX=360102 GN=YPA_2172 PE=3 SV=1 | cell membrane |
| tr\|A0A0E1NQW6\|A0A0E1NQW6_YERPA | SAM-dependent methyltransferase OS=Yersinia pestis bv. Antiqua (strain Antiqua) OX=360102 GN=YPA_2533 PE=4 SV=1 | extracellular membrane |
| tr\|A0A0E8XIA0\|A0A0E8XIA0_9GAMM | LysR family transcriptional regulator OS=Yersinia wautersii OX=1341643 GN=lysR8 PE=3 SV=1 | cytoplasm |
| tr\|A0A0H2W204\|A0A0H2W204_YERPE | Putative exported protein OS=Yersinia pestis OX=632 GN=YP_0691 PE=3 SV=1 | extracellular membrane |
| tr\|A0A0E1NT26\|A0A0E1NT26_YERPA | UvrABC system protein A OS=Yersinia pestis bv. Antiqua (strain Antiqua) OX=360102 GN=uvrA PE=3 SV=1 | cell membrane |
| tr\|A0A0U1QU39\|A0A0U1QU39_YERP3 | IS10， transposase OS=Yersinia pseudotuberculosis serotype O:1b (strain IP 31758) OX=349747 GN=YpsIP31758_2541 PE=4 SV=1 | cell membrane |
| tr\|A0A380Q9L6\|A0A380Q9L6_YERPU | Predicted phage phi-C31 gp36 major capsid-like protein OS=Yersinia pseudotuberculosis OX=633 GN=NCTC8580_02698 PE=4 SV=1 | cell membrane，extracellular membrane |
| tr\|A0A0E1NQC2\|A0A0E1NQC2_YERPA | L-threonine ammonia-lyase OS=Yersinia pestis bv. Antiqua (strain Antiqua) OX=360102 GN=YPA_2996 PE=4 SV=1 | cytoplasm |
| tr\|A0A0T9R9J5\|A0A0T9R9J5_9GAMM | IS1329 transposase B OS=Yersinia similis OX=367190 GN=tnpB_1 PE=4 SV=1 | cytoplasm |
| tr\|A0A0T9NZZ1\|A0A0T9NZZ1_9GAMM | Membrane protein OS=Yersinia similis OX=367190 GN=ERS008667_00443 PE=4 SV=1 | cell membrane |
| tr\|A0A0E1NXL2\|A0A0E1NXL2_YERPA | Alginate lyase OS=Yersinia pestis bv. Antiqua (strain Antiqua) OX=360102 GN=YPA_2976 PE=4 SV=1 | extracellular membrane |

**Table 1-3**: rOMVyp2unique protein

| Protein name | Protein Information | Subcellular localization |
| --- | --- | --- |
| tr\|A0A0E1NT12\|A0A0E1NT12_YERPA | Succinate dehydrogenase flavoprotein subunit OS=Yersinia pestis bv. Antiqua (strain Antiqua) OX=360102 GN=YPA_0589 PE=3 SV=1 | cytoplasm |
| tr\|A0A0T9RIT1\|A0A0T9RIT1_9GAMM | Elongation factor Tu OS=Yersinia similis OX=367190 GN=tufA_2 PE=3 SV=1 | cytoplasm |
| tr\|A0A0T9PH46\|A0A0T9PH46_9GAMM | Peptidase B OS=Yersinia similis OX=367190 GN=pepB PE=3 SV=1 | cytoplasm |
| tr\|A0A0E1NUM7\|A0A0E1NUM7_YERPA | Periplasmic serine endoprotease DegP-like OS=Yersinia pestis bv. Antiqua (strain Antiqua) OX=360102 GN=YPA_2881 PE=3 SV=1 | extracellular membrane |
| tr\|A0A0E1NQ95\|A0A0E1NQ95_YERPA | ABC-transporter outer membrane component OS=Yersinia pestis bv. Antiqua (strain Antiqua) OX=360102 GN=YPA_3129 PE=3 SV=1 | extracellular membrane |
| tr\|A0A250MDC5\|A0A250MDC5_YERPU | TerC OS=Yersinia pseudotuberculosis OX=633 PE=3 SV=1 | cell membrane |
| tr\|A0A0E1NM81\|A0A0E1NM81_YERPA | Cysteine synthase OS=Yersinia pestis bv. Antiqua (strain Antiqua) OX=360102 GN=YPA_2182 PE=3 SV=1 | cytoplasm |
| tr\|A0A0E8XI98\|A0A0E8XI98_9GAMM | Chaperone protein DnaK OS=Yersinia wautersii OX=1341643 GN=dnaK PE=2 SV=1 | cytoplasm |
| tr\|A0A380Q4V6\|A0A380Q4V6_YERPU | LPS-assembly protein LptD OS=Yersinia pseudotuberculosis OX=633 GN=lptD PE=3 SV=1 | extracellular membrane |
| tr\|A0A0E8XNC1\|A0A0E8XNC1_9GAMM | DNA-directed RNA polymerase subunit beta OS=Yersinia wautersii OX=1341643 GN=rpoB PE=3 SV=1 | cytoplasm |
| tr\|A0A0T9PVF1\|A0A0T9PVF1_9GAMM | Quinone-dependent D-lactate dehydrogenase OS=Yersinia similis OX=367190 GN=dld PE=3 SV=1 | cytoplasm |
| tr\|A0A0E8XG14\|A0A0E8XG14_9GAMM | Dihydroorotate dehydrogenase (quinone) OS=Yersinia wautersii OX=1341643 GN=pyrD PE=3 SV=1 | cytoplasm |
| tr\|A0A0E8XLP6\|A0A0E8XLP6_9GAMM | Serine endoprotease OS=Yersinia wautersii OX=1341643 GN=degS PE=3 SV=1 | extracellular membrane |
| tr\|A0A0E1NU51\|A0A0E1NU51_YERPA | Outer membrane protein assembly factor BamD OS=Yersinia pestis bv. Antiqua (strain Antiqua) OX=360102 GN=bamD PE=3 SV=1 | extracellular membrane |
| tr\|A0A0E1NM49\|A0A0E1NM49_YERPA | L，D-transpeptidase OS=Yersinia pestis bv. Antiqua (strain Antiqua) OX=360102 GN=YPA_1743 PE=3 SV=1 | extracellular membrane |
| tr\|A0A0E8XPC4\|A0A0E8XPC4_9GAMM | 50S ribosomal protein L19 OS=Yersinia wautersii OX=1341643 GN=rplS PE=3 SV=1 | cytoplasm |
| tr\|A0A0E8XJ33\|A0A0E8XJ33_9GAMM | Cation/acetate symporter ActP OS=Yersinia wautersii OX=1341643 GN=actP PE=3 SV=1 | cell membrane |
| tr\|A0A0E8XH25\|A0A0E8XH25_9GAMM | Membrane-bound lytic murein transglycosylase A OS=Yersinia wautersii OX=1341643 GN=mltA PE=4 SV=1 | extracellular membrane |
| tr\|A0A0E1NRD7\|A0A0E1NRD7_YERPA | Probable peptidoglycan glycosyltransferase FtsW OS=Yersinia pestis bv. Antiqua (strain Antiqua) OX=360102 GN=ftsW PE=3 SV=1 | cell membrane |
| tr\|A0A0E1NRK8\|A0A0E1NRK8_YERPA | Uridine phosphorylase OS=Yersinia pestis bv. Antiqua (strain Antiqua) OX=360102 GN=YPA_0238 PE=3 SV=1 | cytoplasm |
| tr\|A0A0E1NPP5\|A0A0E1NPP5_YERPA | Dihydrolipoyllysine-residue succinyltransferase component of 2-oxoglutarate dehydrogenase complex OS=Yersinia pestis bv. Antiqua (strain Antiqua) OX=360102 GN=YPA_0592 PE=3 SV=1 | cytoplasm |
| tr\|A0A0E8XLW9\|A0A0E8XLW9_9GAMM | Lipoprotein NlpI OS=Yersinia wautersii OX=1341643 GN=nlpI PE=4 SV=1 | cell membrane，cytoplasm |
| tr\|A0A0E1NSQ7\|A0A0E1NSQ7_YERPA | Efflux pump membrane transporter OS=Yersinia pestis bv. Antiqua (strain Antiqua) OX=360102 GN=YPA_2628 PE=3 SV=1 | cell membrane |
| sp\|Q0WCK7\|BAMC_YERPE | Outer membrane protein assembly factor BamC OS=Yersinia pestis OX=632 GN=bamC PE=3 SV=1 | cell membrane，extracellular membrane |
| tr\|A0A0T9RJJ4\|A0A0T9RJJ4_9GAMM | Beta-galactosidase OS=Yersinia similis OX=367190 GN=lacZ PE=3 SV=1 | cytoplasm |
| tr\|Q5ND80\|Q5ND80_9GAMM | WbcP protein OS=Yersinia sp. A125 KOH2 OX=595656 GN=wbcP PE=3 SV=1 | cytoplasm |
| tr\|A0A0E8XPN5\|A0A0E8XPN5_9GAMM | FaeA-like protein OS=Yersinia wautersii OX=1341643 GN=ERS008478_03630 PE=4 SV=1 | cell membrane，cytoplasm |
| tr\|A0A0T9NRQ7\|A0A0T9NRQ7_9GAMM | Aconitate hydratase B OS=Yersinia similis OX=367190 GN=acnB PE=3 SV=1 | cytoplasm |
| tr\|A0A0E1NW79\|A0A0E1NW79_YERPA | NADP-dependent malic enzyme OS=Yersinia pestis bv. Antiqua (strain Antiqua) OX=360102 GN=YPA_2222 PE=3 SV=1 | cytoplasm |
| tr\|A0A0E1NMT8\|A0A0E1NMT8_YERPA | Putative ABC transporter， ATP-binding protein OS=Yersinia pestis bv. Antiqua (strain Antiqua) OX=360102 GN=YPA_2955 PE=4 SV=1 | cell membrane |
| tr\|A0A0E1NP34\|A0A0E1NP34_YERPA | ATP-dependent DNA helicase DinG OS=Yersinia pestis bv. Antiqua (strain Antiqua) OX=360102 GN=dinG PE=3 SV=1 | cytoplasm |
| tr\|A0A0E1NVC4\|A0A0E1NVC4_YERPA | Glycerophosphoryl diester phosphodiesterase OS=Yersinia pestis bv. Antiqua (strain Antiqua) OX=360102 GN=YPA_0195 PE=4 SV=1 | cytoplasm |
| tr\|A0A0H3B7D2\|A0A0H3B7D2_YERPY | Sulfite reductase [NADPH] flavoprotein alpha-component OS=Yersinia pseudotuberculosis serotype O:3 (strain YPIII) OX=502800 GN=cysJ PE=3 SV=1 | cytoplasm |
| tr\|A0A6M3GA28\|A0A6M3GA28_9CAUD | Neck protein OS=Yersinia phage vB_YepM_ZN18 OX=2691085 PE=4 SV=1 | extracellular membrane |
| tr\|A0A2C8CUX9\|A0A2C8CUX9_YERPE | IncH1 plasmid conjugative transfer protein Orf9 OS=Yersinia pestis OX=632 GN=P2180_164 PE=4 SV=1 | cell membrane，extracellular membrane |
| tr\|A0A0E1NNN5\|A0A0E1NNN5_YERPA | Transcription termination/antitermination protein NusG OS=Yersinia pestis bv. Antiqua (strain Antiqua) OX=360102 GN=nusG PE=3 SV=1 | cytoplasm |
| tr\|A0A0E1NN65\|A0A0E1NN65_YERPA | D-alanyl-D-alanine carboxypeptidase OS=Yersinia pestis bv. Antiqua (strain Antiqua) OX=360102 GN=YPA_0611 PE=4 SV=1 | cell membrane |
| tr\|A0A0H3B1U1\|A0A0H3B1U1_YERPY | Helicase ATP-binding domain-containing protein OS=Yersinia pseudotuberculosis serotype O:3 (strain YPIII) OX=502800 GN=YPK_1197 PE=4 SV=1 | cytoplasm |
| tr\|A0A0E8XG26\|A0A0E8XG26_9GAMM | UPF0283 membrane protein ERS008478_01183 OS=Yersinia wautersii OX=1341643 GN=ycjF PE=3 SV=1 | cytoplasm |
| tr\|A0A8K0ZRK3\|A0A8K0ZRK3_9CAUD | Flavodoxin-like domain-containing protein OS=Yersinia phage PYps3T OX=2801357 GN=ORF054 PE=4 SV=1 | cell membrane，extracellular membrane |
| tr\|A0A0T9Q3Y8\|A0A0T9Q3Y8_9GAMM | DNA protection during starvation protein OS=Yersinia similis OX=367190 GN=dps PE=3 SV=1 | cytoplasm |
| tr\|A0A0T9PJ48\|A0A0T9PJ48_9GAMM | Alkaline phosphatase OS=Yersinia similis OX=367190 GN=phoA PE=3 SV=1 | extracellular membrane |
| tr\|A0A0U1QUL5\|A0A0U1QUL5_YERP3 | Putative type I restriction-modification system， S subunit OS=Yersinia pseudotuberculosis serotype O:1b (strain IP 31758) OX=349747 GN=YpsIP31758_3537 PE=3 SV=1 | cell membrane |
| tr\|A0A0U1QT73\|A0A0U1QT73_YERP3 | Putative type IV secretion system protein PilO2 OS=Yersinia pseudotuberculosis serotype O:1b (strain IP 31758) OX=349747 GN=YpsIP31758_A0061 PE=4 SV=1 | cell membrane，extracellular membrane |
| tr\|A0A0E1NSJ7\|A0A0E1NSJ7_YERPA | Cellulase OS=Yersinia pestis bv. Antiqua (strain Antiqua) OX=360102 GN=YPA_3825 PE=3 SV=1 | extracellular membrane |
| tr\|A0A0E8XGT3\|A0A0E8XGT3_9GAMM | Sec-independent protein translocase protein TatB OS=Yersinia wautersii OX=1341643 GN=tatB PE=3 SV=1 | cell membrane，cytoplasm |
| tr\|A0A380Q4K3\|A0A380Q4K3_YERPU | Efflux pump membrane transporter OS=Yersinia pseudotuberculosis OX=633 GN=acrB_1 PE=3 SV=1 | cell membrane |
| tr\|A0A0E1NY46\|A0A0E1NY46_YERPA | Putative hydrolase OS=Yersinia pestis bv. Antiqua (strain Antiqua) OX=360102 GN=YPA_3326 PE=4 SV=1 | cytoplasm |
| tr\|A0A0T9RNU1\|A0A0T9RNU1_9GAMM | Peptidoglycan-binding protein OS=Yersinia similis OX=367190 GN=ERS008667_04166 PE=4 SV=1 | cell membrane，extracellular membrane |
| tr\|A0A0T9PT79\|A0A0T9PT79_9GAMM | p22AR C-terminal domain OS=Yersinia similis OX=367190 GN=ERS008667_01582 PE=4 SV=1 | cell membrane |
| tr\|A0A0E1NVD9\|A0A0E1NVD9_YERPA | Protein translocase subunit SecE OS=Yersinia pestis bv. Antiqua (strain Antiqua) OX=360102 GN=secE PE=3 SV=1 | cell membrane |
| tr\|A0A6M3G8W5\|A0A6M3G8W5_9CAUD | DNA-directed DNA polymerase OS=Yersinia phage vB_YepM_ZN18 OX=2691085 PE=3 SV=1 | cytoplasm |
| tr\|A0A0E1NP10\|A0A0E1NP10_YERPA | Putative sugar transporter OS=Yersinia pestis bv. Antiqua (strain Antiqua) OX=360102 GN=YPA_2027 PE=4 SV=1 | cell membrane |
| tr\|A0A3G5KN85\|A0A3G5KN85_YERPU | Tail fiber protein OS=Yersinia pseudotuberculosis OX=633 GN=EGX52_08710 PE=4 SV=1 | cell membrane，extracellular membrane |
| tr\|A0A0E1NMV9\|A0A0E1NMV9_YERPA | Putative lipoprotein OS=Yersinia pestis bv. Antiqua (strain Antiqua) OX=360102 GN=YPA_2354 PE=4 SV=1 | cytoplasm |
| tr\|A0A0E1NPN4\|A0A0E1NPN4_YERPA | Sugar transport ATP-binding protein OS=Yersinia pestis bv. Antiqua (strain Antiqua) OX=360102 GN=YPA_0412 PE=4 SV=1 | cell membrane |
| tr\|A0A0E1NP31\|A0A0E1NP31_YERPA | Putative DEAD box helicase family protein OS=Yersinia pestis bv. Antiqua (strain Antiqua) OX=360102 GN=YPA_0983 PE=4 SV=1 | cytoplasm |
| tr\|A0A0H2W099\|A0A0H2W099_YERPE | Type IV secretory pathway VirB2 component OS=Yersinia pestis OX=632 GN=virB2 PE=4 SV=1 | cell membrane |
| tr\|A0A0E8XLP1\|A0A0E8XLP1_9GAMM | 30S ribosomal protein S9 OS=Yersinia wautersii OX=1341643 GN=rpsI PE=3 SV=1 | cytoplasm，extracellular membrane |
| tr\|A0A0H2W5X1\|A0A0H2W5X1_YERPE | Galactose operon repressor OS=Yersinia pestis OX=632 GN=galR2 PE=4 SV=1 | cytoplasm |
| tr\|A0A250MF63\|A0A250MF63_YERPU | Magnesium transporter OS=Yersinia pseudotuberculosis OX=633 PE=4 SV=1 | extracellular membrane |
