# Supplementary material for: Effects of Yersinia pseudotuberculosis outer membrane vesicles on Pseudomonas aeruginosa antigens immune response
Source: PLoS One. 2024 Dec 20;19(12):e0310652. doi: 10.1371/journal.pone.0310652 (PMC11661608; doi:10.1371/journal.pone.0310652)
Supplement: S1 Raw image — (DOC) [file pone.0310652.s001.doc]

**S1_raw_image**

**Figure S1 A** Raw images used for analysis. SDS-PAGE electrophoresis diagram.

**Figure S1 B** Raw images used for analysis. SDS-PAGE electrophoresis diagram.

**Figure S1 C.** Raw images used for analysis.Western blot diagram.

**Figure S1D.** Raw images used for analysis.Western blot diagram.

**Figure S1E** Raw images used for analysis. Agarose gel electrophoresis diagram

**Figure S1F** Raw images used for analysis. Agarose gel electrophoresis diagram

**Figure S1 G** Raw images used for analysis. SDS-PAGE electrophoresis diagram.

**Figure S1 H** Raw images used for analysis. SDS-PAGE electrophoresis diagram.

**Figure S1 I** Raw images used for analysis.Western blot diagram.

**Figure S1 J** Raw images used for analysis.Western blot diagram.
